# Deciphering the Protein Phosphorylation Dynamics Triggered by Seconds of Force Stimulation

**DOI:** 10.1016/j.mcpro.2026.101532

**Published:** 2026-02-19

**Authors:** Nan Yang, Sunny Sing Pun, Emily Oi Ying Wong, Shuaijian Dai, Xiaoting Li, Manhin Leung, Al Burlingame, Zhi-Yong Wang, Minglei Yang, Yinglin Lu, Yuxing An, Yage Zhang, Zhu Yang, Weichuan Yu, Ning Li

**Affiliations:** 1 Division of Life Science, The Hong Kong University of Science and Technology, Hong Kong, Hong Kong SAR, China; 2 Department of Pharmaceutical Chemistry, University of California San Francisco, San Francisco, California, USA; 3 Department of Plant Biology, Carnegie Institution for Science, Stanford, USA; 4 Department of Thoracic Surgery, Ningbo No. 2 Hospital, Ningbo, Zhejiang Province, China; 5 Institute of Nanfan and Seed Industry, Guangdong Academy of Sciences, Guangzhou, Guangdong, China; 6 School of Biomedical Engineering, Shenzhen University Medical School, Shenzhen University, Shenzhen, Guangdong, China; 7 Guangdong Key Laboratory of Biomedical Engineering, Shenzhen University, Shenzhen, Guangdong, China; 8 State Key Laboratory of Environmental and Biological Analysis, Department of Biology, Hong Kong Baptist University, Hong Kong, Hong Kong SAR, China; 9 Department of Electronic and Computer Engineering, The Hong Kong University of Science and Technology, Hong Kong, Hong Kong SAR, China; 10 AI-X Open Laboratory, HKUST Collaborative Innovation Research Institute, Shenzhen, China

**Keywords:** Arabidopsis, 4C quantitative phosphoproteomics, force signaling, gravity-regulated phosphoprotein 1 (GREPH1), seconds of phosphorylation events, lkelihood of interaction and function evaluation (LIFE) score

## Abstract

Plants perceive mechanical forces through phosphosignaling networks, but their relationship with gravity signaling remains elusive. To dissect gravity force signaling components, we performed SILIA-based phosphoproteomics on Arabidopsis aerial organs subjected to 20-s inversion or 30-s gravistimulation, identifying 2,733 and 2,878 phosphoproteins, respectively. Quantitative analysis revealed 34 significantly regulated phosphoproteins specific to inversion and 52 specific to gravistimulation. Inversion-specific phosphoproteins, associated with the initial calcium code, likely mediate calcium signals through EF-hand proteins, CPK1, and calmodulin-interacting proteins, potentially intersecting with receptor-like kinase-initiated MAPK cascades via RAF15 and MKK1/2 to induce gravitropic responses. Gravistimulation-specific phosphoproteins, linked to the secondary calcium code, function in calcium signaling/homeostasis (ACA8, ZAC, IQD2, ANNAT1), membrane vesicle trafficking (ABCG36, ARF-GAP8), and lipid signaling (PIP5K8/9), supporting auxin transport and stress signal transduction. Immunoblot validation confirmed treatment-associated phosphosites pS108-PATL3 and pS107-TREPH2, along with inversion-specific pS1145-ATEH2, exhibiting stem-specific phosphorylation enhancement and force-discriminatory responses. Functional analysis identified the integrin-like protein GREPH1 as a key gravitropism regulator, with greph1 mutants displaying reduced inflorescence stem gravicurvature. Notably, hyperphosphorylation of pS107-TREPH2 and pS1145-ATEH2 peaked at 20 to 50 s in greph1 mutants but persisted from 20 s to 2 h in WT plants. These findings establish a stem-enriched phosphorylation code for gravity force discrimination, with GREPH1 modulating spatiotemporal phosphoprotein dynamics and shoot gravicurvature, potentially functioning as a receptor reminiscent of sedimenting plastids.

## Highlights

• Phosphoproteomics performed on 20-s inversion– and 30-s gravistimulation–treated Arabidopsis.
• PTM proteomics identified 2,735 and 2,885 phosphoproteins, from two treatments, respectively.
• Identified were 34- and 52-specific phosphoproteins from two treatments, respectively.
• The rotation-specific signals are mediated by phosphor-relays through CPK1, RAF15 kinases.
• The gravity-specific p-proteins include trafficking, PIN, and auxin transport proteins.
• GREPH1 regulates phosphorylation of TREPH2, ATEH2, and PALT3.7.
• GREPH1 gene plays a role in shoot gravitropism.

## Graphical Abstract

**Figure:**
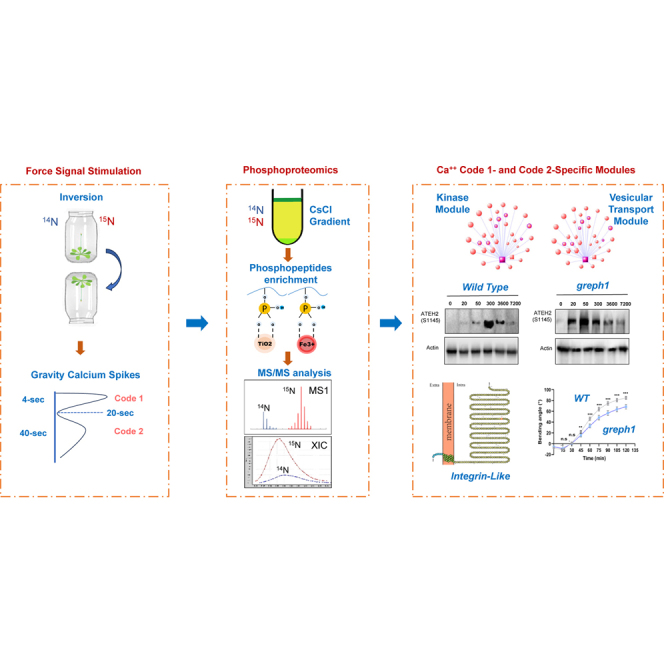

## In Brief

The *SILIA*-based phosphoproteomics identified 34 and 52 significantly regulated phosphoprotein groups from inversion- and gravistimulation force-treated *Arabidopsis*, respectively. The 20 s inversions quickly induce phosphorylation of EF-hand proteins, calmodulin-interacting proteins, CPK1, RAF15 and MKK1/2 kinases, whereas the phosphoproteins from 30-s upside-down hold samples have the unique functions in Ca^2+^ signaling/homeostasis, vesicle trafficking, lipid signaling, and PIN protein/auxin molecule transport for force signal transduction. Functional validation showed the integrin-like protein GREPH1 acting as a key regulator of gravitropism, modulating spatiotemporal phosphoproteome dynamics.

Plants have evolved adaptive responses to environmental cues through tropisms, including phototropism (1), thigmotropism (2), hydrotropism (3), and gravitropism (4, 5, 6). Gravitropism specifically enables downward root growth (positive) and upward shoot growth (negative) to optimize development in response to gravity (7). This directional growth involves sequential steps: gravity signal perception, biochemical signal conversion, signal transduction, and asymmetric organ growth (8, 9, 10). Crucially, plants exhibit distinct mechanisms for gravistimulation (angular displacement <180°) and inversion (180° reorientation) (11).

Two primary hypotheses explain gravity perception: the starch-statolith model (amyloplast sedimentation) dominates current research (12, 13), while the protoplast pressure hypothesis (membrane tension) provides an alternative (14, 15). Specialized statocytes in root columella and shoot endodermis contain starch-granule plastids (amyloplasts) for gravity-sensing (13, 16, 17). These cells show stimulus-dependent divergence: gravistimulation engages amyloplast-dependent lazy gene family protein (LZY (9, 18), polarization, while inversion might activate membrane-bound mechanosensory receptors (*e.g*. adhesion G protein–coupled receptors) before amyloplast sedimentation (11, 19, 20). Key cellular distinctions include vacuolar differences between statocyte types (8). Arabidopsis shoot gravitropism mutants (*sgr2*, *sgr3*, *sgr4*, *sgr6*) demonstrate the vacuole's critical role (21, 22, 23). Mechanistic models propose either actin-mediated mechanotransduction of amyloplast sedimentation forces (24) involving calcium channels (25) or amyloplast positioning as inclination sensors (11), though calcium's specific function requires clarification (26). Calcium signatures diverge markedly: an upside-down inversion generates a biphasic calcium spike within 50 s, which includes a single initial mechanical (probably centrifugal force-specific) calcium transient, peaking at 4 s of inversion followed by a severe decay by 20 s and a gravity force-specific secondary calcium spike, peaking at 40 s (25).

Auxin redistribution drives asymmetric growth via Pin-formed 3/7 (PIN3/7) transporter polarization (27, 28, 29), regulated by statocyte-expressed *LZY* gene family proteins (30) interacting with Regulator of Chromatin Condensation 1–Like domains (31, 32). Force stimulus type determines auxin dynamics: gravistimulation enables robust PIN3 relocalization and sustained asymmetry, whereas inversion might cause erratic gradients due to mechanical stress interference (9). Upstream regulators like Rice Prostrate Growth 1 influence *LZY1* activity (33), but still the early signal reception mechanism remains unresolved.

Research since Darwin's initial documentation employs diverse methods to elucidate mechanistic gravitropism (34). Forward genetics identified key mutants (*sgr1–sgr9*) and validated endodermal function in dicot shoots (35, 36, 37, 38). Genetic evidence confirms mechanistic divergence: starchless mutants retain inversion responses but lack gravistimulated curvature (39). *SGR9* regulates amyloplast-actin dynamics as a plastid-localized E3 ligase (39). Omics approaches revealed comprehensive responses. For examples, the transcriptomics identifies shared gravity/mechanical response genes (40, 41) and *LZY1*-related expression changes (42, 43), while the proteomics performed on the minutes and hours of gravistimulated plant organs detected cytoskeletal/Ca^2+^ alterations (44) and Glutathione S-Transferase Phi(F) class 9/heat shock protein 81-2 (HSP81-2) (45) as well as adenosine kinase 1 upregulation being upstream of roles of PIN3 protein (46).

Protein phosphorylation is a biochemical event, the most ubiquitous type of posttranslational modification (PTM) occurred in cells, influencing virtually every fundamental cellular process in response to both developmental and environmental cues (47). The time course of phosphorylation upon a signal induction can reveal dynamic and causal relationships among molecular components in signal transduction (48). Protein phosphorylation events occurred rapidly within seconds (49). Pin phosphorylation is sufficient to regulate auxin transport (50). The 40 s of touch-induced instant protein phosphorylation could be measured by quantitative phosphoprotemics (51, 52, 53). By the same phosphoproteomics approach, we have revealed gravity stimulus-specific phosphoproteins: Patellin 3 (PATL3) phosphorylation marks gravistimulation while Phototropin 1 is involved within 150 s of gravitropic response of the loss-of-function ethylene response 1 to 6 mutant (*etr1-6*, (54).

Building on the evidence of the biphasic calcium spike signaling during gravitropic response (25), we hereby employ the 4C quantitative phosphoproteomics to investigate both 20 s of multiple inversion stimulation (simplified as inversion)- and 30 s of 180° upside-down hold stimulation (simplified as gravistimulation)-triggered phosphoproteins. Based on bioinformatic investigation of two large groups of inversion and gravistimulation significantly regulated phosphoproteins and immunoblot analysis of phosphorylation patterns, PATL3, touch-regulated phosphoprotein 2 (TREPH2), and *Arabidopsis thaliana* EF-hand protein 2 (ATEH2) have been confirmed as distinct mechano-stimulation–specific protein PTM biomarkers. Regulation of the phosphorylation of these biomarkers by Gravity REgulated PHosphoprotein 1 (*GREPH1*) gene validated it is role in shoot gravitropism and identified a novel early force-sensing and force-signaling component in gravity signal transduction pathways.

## Experimental Procedures

### Experimental Design and Statistical Rationale

**Fig. 1:**
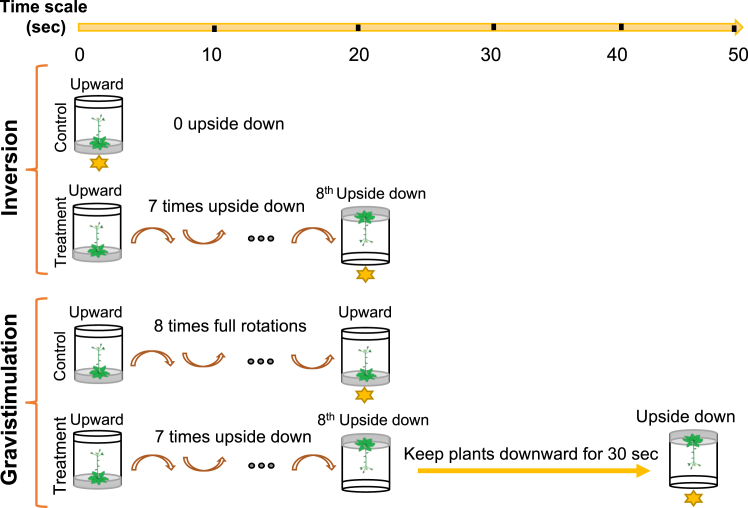
**Inversion and gravistimulation experiments.** Inversion experiment, control: tissues collected at upright position (0 s, no inversion). Treatment: samples collected at 20 s after undergoing eight consecutive 180° inversions (upside-down rotations; Videos 1 and 2). Gravistimulation experiment, control: samples collected after eight complete 360° rotations, ending in upright position. Treatment: samples subjected to seven full rotations and maintained at the upside-down (eighth rotation) position, which was followed by 30 s pause in the same position (Video 3 and 4). *Yellow bars* indicate treatment durations (s). *Star symbols* (★) mark time points when aerial tissues were flash-frozen in liquid nitrogen and collected from culture vessels (see Videos for detailed methods) (Supplemental Table S1).

To map phosphoproteomic dynamics involved in early gravitropic response, including sensing of gravity signals and the initial gravity signal transduction, the stable isotope labeling in *Arabidopsis* (SILIA)-based quantitative PTM proteomics (52, 53, 55, 56) was adopted. Based on the findings of Toyota *et al*. (25), the simple inversion of plant (145°–180° of upside down) could elicit transient biphasic cytoplasmic calcium concentration peaks, a peak at 4 s and the other at 40 s after the change in gravity vector (25). The first calcium spike was speculated to result from the centrifugal force and gravity force signal, while the second spike from the gravity vector changes alone. The higher speed of rotation could only induce a higher initial calcium spike but not the second gravity-specific calcium transient, suggesting that a single inversion of plant can elicit two types of mechanical signals on plant organs. To distinguish the effects of inversion forces from that of the gravity force on phosphoproteomic landscape changes, we designed two sets of mechano-stimulation experiments (Fig. 1). One set of experiments focused on the phosphoproteomic analysis on inversion stimulation, whereas the other set emphasized on phosphoproteomic analysis on gravistimulation.

The *SILIA*-based and 4C quantitative phosphoproteomics workflow applied in studying the phosphoproteins regulated by both inversion forces and gravity force (Supplemental Fig. S1) include the following steps: first C, *in vivo* chemical labeling (^15^N and ^14^N) of the total cellular proteins of *Arabidopsis* plants (*SILIA*, (55); second C, chromatographic enrichment and LC-MS/MS analysis of the phosphopeptides; third C, computational identification, quantification of the mass spectrometry (MS) data, as well as a comprehensive bioinformatic analysis of the quantitative phosphoproteomic results; fourth C, confirmation and validation of the *SILIA*-based quantitative PTM phosphoproteomics results using molecular biology and molecular genetics methods.

To identify more nonredundant (or unique) phosphopeptides, we separated the total protein into both membrane (M) and cytoplasmic (C) fractions using caesium chloride (CsCl) gradient centrifugation in both inversion stimulation (20 s) and gravistimulation (30 s) experiments. The tissue sample collection, protein and peptide preparation, and phosphopeptide enrichment, were summarized in Supplemental Table S1 for both experiments. Mascot-searched (57) MS data of inversion stimulation (inversion-C and inversion-M) and gravistimulation (gravity-C and gravity-M) fractions were summarized in Supplemental Tables S2 and S3. The log_2_ ratios of the extracted ion chromatography intensities of treatment (case) over control unique PTM site patterns (UPSPs) were generated using a previously published *SILIA*-based quantification method, stable isotope-based quantitation (*SQUA*), (Supplemental Tables S4–S7) (53, 58) to identify inversion (rotational force plus gravity force) forces- and gravity force–regulated phosphopeptides.

### Plant Materials and CRISPR KO Mutants

Seeds from *Arabidopsis thaliana* ecotype *Col-0* and T-DNA insertional mutant lines *patl3* (SALK_093994, insertion in AT1G72160,) (59), *ateh2* (At1g21630, Salk_0922023), and *treph2* (CS850265, insertion in AT4G26070) (Supplementals Figs. S7 and S8 (52, 54) were purchased from *Arabidopsis* Biological Resource Center. Seeds of *A. thaliana Col-0* ecotype harboring the Pro35S::Aequorin (*AEQ*) recombinant gene was a gift from Dr Knight (60). Using *AEQ* transgenic plants (AEQ/Col-0) as the genotype background, a group of candidate genes were knocked out using the CRISPR/CAS9 method (Genovo Bio) (61). This group of CRISPR/Cas9 KO plants was screened for the bending defect upon the gravity vector alteration. A mutant named *greph1* was then selected to verify its gravitropic response of the hypercotyl following gravistimulation. Both *greph1-1* and *greph1-2* lines were obtained from the KO experiments performed on the *AEQ* transgenic plants. The genotyping results are shown in Supplemental Fig. S14.

### Plant Growth Medium and SILIA

All unspecified chemicals were purchased from Sigma. Seeds of the *Arabidopsis AEQ* transgenic plants were sterilized with 0.1% Triton X-100 and 30% bleach in 70% ethanol, and after discarding the buffer, sterile water was added to wash the seeds at least four times. Seeds covered with aluminium foil were vernalized by soaking in sterile double distil water at 4 °C for 4 to 7 days. In the case of Stable Isotope Labelling In *Arabidopsis* (*SILIA*)-based quantitative phosphoproteomic experiments, the plant growth medium contains 5 mM NH_4_NO_3_ (^15^N or ^14^N), 18.8 mM KNO_3_ (^15^N or ^14^N), 100 μM MnSO_4_·7H_2_O, 100 μM NaEDTA·2H_2_O, 1 μM Na_2_MoO_4_·2H_2_O, 100 μM H_3_BO_3_, 100 μM FeSO_4_·7H_2_O, 1.25 mM KH_2_PO_4_, 3 mM CaCl_2_·2H_2_O, 0.1 μM CoCl_2_.5H_2_O, 30 μM ZnSO_4_·2H_2_O, 0.1 μM CuSO_4_·5H_2_0, 1.5 mM MgSO_4_·H_2_O, 10 mg/l thiamine HCl, 100 mg/l myo-inositol, 1 mg/l pyridoxine, 1 mg/l nicotinic, 10 g/l sucrose, and 8 g/l agar (55). The 50 ml of autoclaved medium was poured into a 7.5 cm by 12.5 cm diameter and 19 cm tall plastic jar. After sewing 15 to 20 seeds on agar surface, all jars were covered with a Hydrophobic Fluoropore Membrane (http://www.shjiafeng.com, Shanghai Jiafeng) to maintain air permeability and sterility. The light intensity applied to the plants was 140 to 180 μE m^−2^ s^−1^, the temperature of the growth chamber was set at 23 ± 1 °C, and the humidity was approximately 35 to 65%.

In the immunoblot and gravicurvature experiments, Murashige and Skoog–based growth medium was used, which contains 10 g/l sucrose, 4.3 g/l Murashige and Skoog medium powder (pH 5.7), and 8 g/l agar.

### Inversion and Gravistimulation Experiments and Tissue Collection for Proteomic and Immunoblot Analysis

The AEQ protein–expressing transgenic plant was chosen to perform the quantitative phosphoproteomic experiment because of the convenience for the subsequent genetic KO experiment to examine the function of the candidate phosphoproteins in gravitropic response. Two groups of *AEQ* plants were grown in either ^15^N isotope- or ^14^N isotope-coded agar media. When the inflorescence lengths of the experimental plants reached 4 to 8 cm long and about 25 days old (the exact day of harvesting depending on the genotype of plant), the plants grown inside plastic jars were treated either with inversion or gravistimulation (two different types of mechanical stimuli) using an in-house manufactured rotation machine (HKUST Laboratory Service, See Video 1, Video 2, Video 3, Video 4). Before the inversion mechanical treatment, the plastic jars were poked from the bottom heel with a red-hot iron bar to produce a hole of 0.5 to 0.7 cm diameter into the internal space of plastic jars where adult plants grow. This hole allowed the vaporized liquid nitrogen to escape during snap-freezing of the upside-down plants (See Video 2).

In the inversion treatment experiment, a pair of the heavy nitrogen (^15^N)- or the light nitrogen (^14^N)-labeled plants growing upward were frozen by the liquid nitrogen poured into jars and harvested as control (C) plant samples (Fig. 1, Video 1), whereas the other pair of ^15^N- or ^14^N-labeled plants were flipped upside down seven rounds on rotation machine (2.4–2.5 s per 360˚ rotation), ending at the upside-down position by 20 ± 1.5 s, which was defined as treated (T) samples (Fig. 1, Video 2). The aerial part of plant organs were immediately submerged inside liquid nitrogen to presumably freeze biochemical activities, including kinase activities, in cells. Consequently, these frozen tissues mixed with dry ices at 1:1 (volume:volume) ratio were ground into fine powders inside a precooled (−20˚) mortar pestle and stored for later protein and peptide preparation.

In the gravistimulation experiment, a pair of ^15^N- (heavy nitrogen, H) and ^14^N- (light nitrogen, L) labeled *AEQ* plants were subjected to 8 times of full rotation stimulation (360˚), ending with an upright position by 20 ± 1.5 s (Fig. 1, Video 3). These plant tissues were frozen by pouring the liquid nitrogen into jars and harvested as the control (C) samples for gravistimulation experiment. The other pair of ^15^N- and ^14^N-labeled *AEQ* plants underwent seven full rounds of rotation (2.4–2.5 s per 360˚ of rotation) and an additional upside down by 20 ± 1.5 s. Consequently, these inverted plants were kept at the upside-down position for additional 30 s to gravistimulate plants Fig. 1, Video 4). This sample was defined as treated (T) samples in gravistimulation experiment. The aerial plant tissues were frozen with liquid nitrogen immediately at the end of 30 ± 1.5 s of the upside-down hold (50 s in total counting from the first inversion, Fig. 1, Video 4). The frozen tissue was consequently scraped from the jar into an aluminium foil bag filled with liquid nitrogen. Details are shown in the video (Video 1, Video 2, Video 3, Video 4). For each biological replicate, we harvested approximately 30 jars of plants and obtained approximately 30 g of tissues from each set of experiment, either light (L)- or heavy(H)- nitrogen labeled control (C) and treated (T) samples for each of three biological repeats. The amount of frozen tissues harvested from each sample is provided in Supplemental Table S1.

The tissue powders of the heavy nitrogen-labeled and inversion-treated (H, T) plants were mixed with the light nitrogen-labeled control tissue (L, C) powders at a ratio of 1.1:1 to create the forward (F) replicate mixture. The reasons for such a ratio of mixing have been justified in previous publication (55). Conversely, the heavy nitrogen-labeled control plant tissue powders (H, C) were mixed with the light-labeled and treated plant tissue powders (L, T) at a ratio of 1.1:1 to obtain the reciprocal (R) replicate mixture. The mixing ratio of both heavy and light nitrogen-coded plant tissues were conducted according to the in-house designed quantitative PTM proteomic methods (52, 53, 55, 56). The labeling efficiency of the plant tissue from Inversion and gravistimulation is 98.3% and 98.2%, respectively.

A total of three biological replicates and six experimental replicates were performed in both sets of inversion and gravistimulation experiments. Thus, a total of six mixtures of treatment (T) and control (C) samples were produced, that is, F1, F2, F3, R1, R2, and R3 (Supplemental Table S1) from either Inversion or Gravistimulation experiment.

In the case of inflorescence gravicurvature analysis, two different *greph1* KO lines (*greph1-1* and *greph1-2*, Supplemental Fig. S14) and *Col-0* lines were used. The plants were grown in Murashige and Skoog agar medium under growth conditions described above. When the inflorescences of bolted plants grew to approximately 2 to 5 cm long (in about 25 days), plants of various genotypes were reoriented in a horizontal position within a dimly lit tent. Photographs of plant inflorescence stem were taken every 15 min to record the gravitropic response of the inflorescence stem over a time course of 120 min, capturing the plants' vertical orientation (54). The gravicurvature induced by gravistimulation was measured using ImageJ (version 1.52i, National Institutes of Health, https://imagej.nih.gov/ij/download/).

To perform gravicurvature analysis of light-grown seedlings of CRISPR/Cas9 KO lines, the seeds of these mutant lines were firstly sterilized and consequently vernalized for 4 days in the darkness. The 70-ml of half-strength MS medium was poured into a square Petri dish (120 mm × 120 mm). The seeds were sown in horizontal lines, with three rows of seeds per dish. The Petri dishes were subsequently placed horizontally under dim light (8 μmol photons m^-2^ s^-1^), with the seed rows aligned in parallel to the direction of the light source and left on growth medium to grow for 4 days. The seedlings were pretreated for 2 days in the dark and subsequently exposed to 10 ppm ethylene. The seedlings were turned 90°, as a gravistimulation. The planted rows were vertical to the surface of the earth. Photographs were taken after 24 h of gravistimulation treatment on the ethylene-pretreated seedlings under the dim green light. The seedling stem curvature was measured by ImageJ.

In addition to those CRISPR-generated mutant lines, *greph1-1* and *greph1-2*, *Col-0*, and *AEQ* transgenic lines were studied for gravitropic response under identical conditions. These experiments were repeated for three times (3 biological replicates, Supplemental Figs. S14 and Fig. 6).

**Fig. 2:**
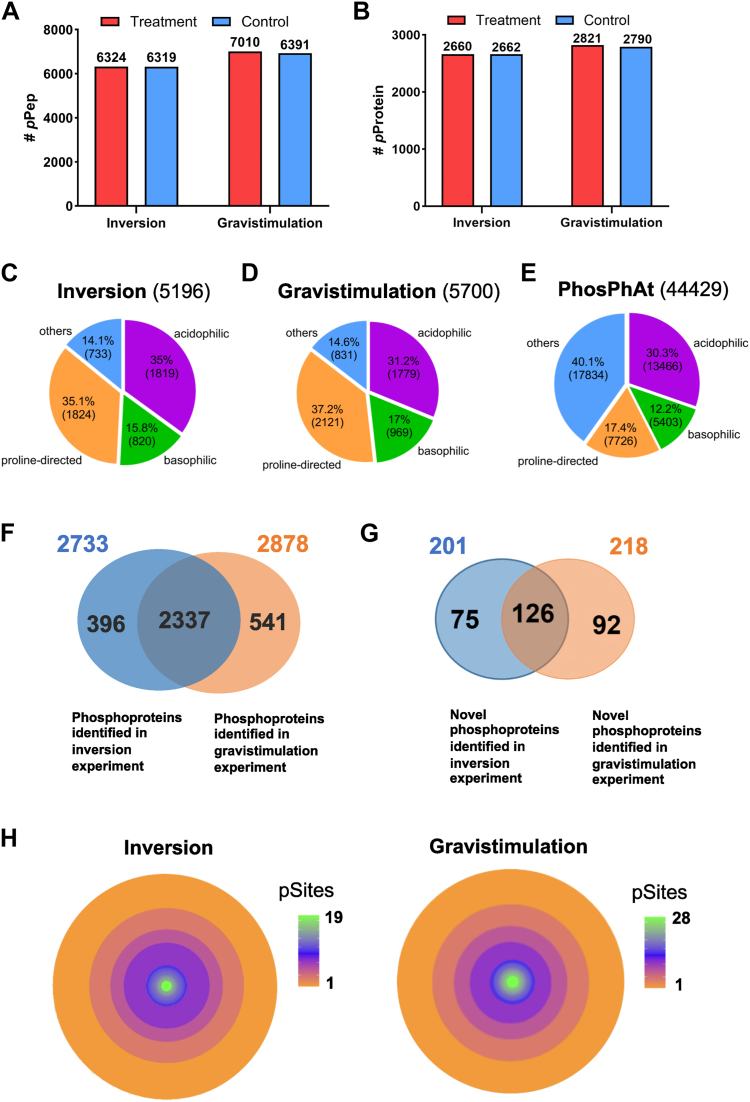
**Phosphoproteomic analysis of Arabidopsis response to inversion and gravistimulation stimulus.***A,* phosphopeptide identification A: bar chart of phosphopeptides (pPeps) identified in the inversion and gravistimulation experiment. *Red*: treatment group; *blue*: control group (Supplemental Tables S2 and S3). *B,* phosphoprotein identification A: Bar chart of phosphoproteins (*p*Proteins) identified in the inversion and gravistimulation experiment. *Red*: treatment group; *blue*: control group (Supplemental Tables S2 and S3). *C–E,* kinase docking site classification phosphorylation sites categorized by kinase recognition motifs in: *C*: inversion experiment. *D,* gravistimulation experiment. *E,* reference database (PhosphAt_20221017) (Supplemental Tables S2 and S3). *F,* a Venn diagram shows the number of identified phosphoproteins from the inversion group (2733, *blue*) and the gravistimulation group (2878, *orange*), respectively (Supplementals Tables S2 and S3). *G,* a Venn diagram presents the number of identified novel phosphoproteins in the Inversion group (201, *blue*) and the gravistimulation group (218, *orange*), respectively. *H,* tree rings diagrams reveal the distribution of phosphoproteins containing different numbers of phosphorylation sites (Supplemental Tables S2 and S3)

**Fig. 3:**
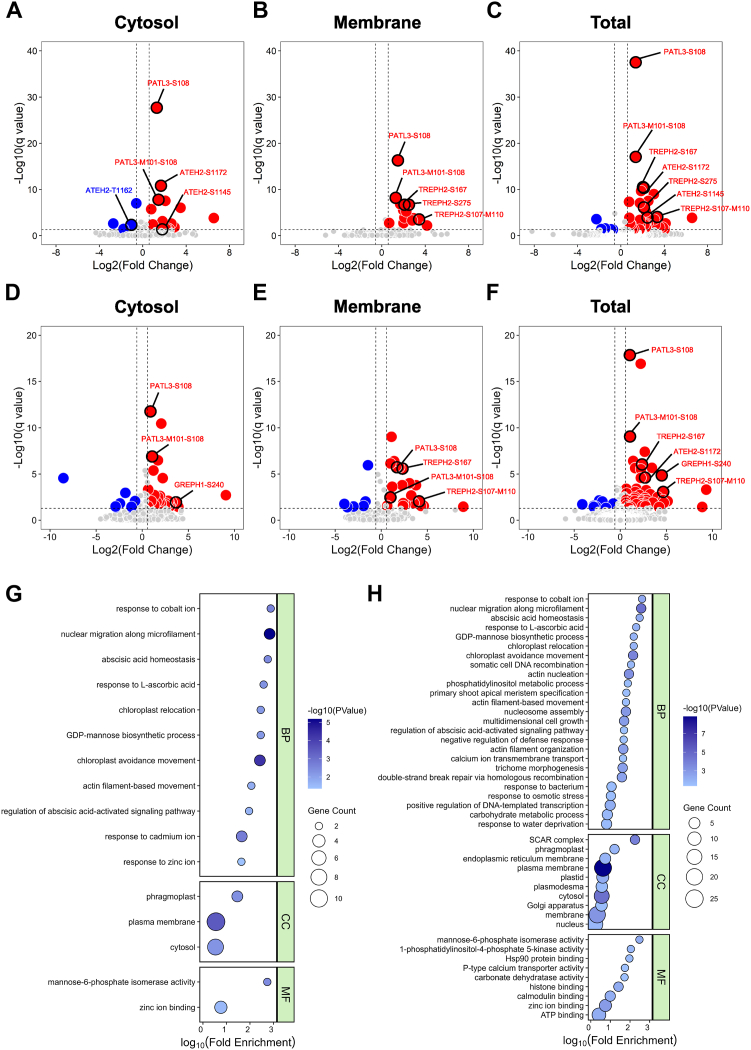
**Quantitative phosphoproteomic analysis of significantly regulated phosphoproteins from the inverted and gravistimulated *Arabidopsis*.***A*–*C,* volcano plots of quantitative phosphoproteomic analysis of plants following inversion stimulation (20 s), quantified from membrane (*A*), cytoplasm (*B*), and total (*C*) fractions. The log2 ratios represent the average binary logarithmic ratios of the phosphopeptide MS1 isotopolog areas, with *p* values derived from the Student's *t* test. These *p* values are further adjusted using the Benjamini–Hochberg procedure to obtain q values. *Vertical and horizontal dashed lines* indicate cutoffs for base 2 logarithm ratios of ±0.6 (fold change = 1.5) and a q value of 0.05, respectively. *Red and blue dots* denote significantly upregulated and downregulated unique PTM site patterns (UPSPs). UPSPs from PTAL3, TREPH2, and ATEH2 are labeled (Supplemental Table S4 d-f). *D–F,* volcano plots of quantitative phosphoproteomic analysis of gravistimulation (30 s), quantified from the membrane (*D*), cytoplasm (*E*), and total (*F*) fractions. The log2 ratios represent the average binary logarithmic ratios of the phosphopeptide MS1 isotopologue areas, with *p* values derived from the Student's *t* test. These *p* values are further adjusted using the Benjamini–Hochberg procedure to obtain q values. *Vertical and horizontal dashed lines* indicate cutoffs for base 2 logarithm ratios of ±0.6 (fold change = 1.5) and a q value of 0.05, respectively. *Red and blue dots* denote significantly upregulated and downregulated unique PTM site patterns (UPSPs). UPSPs from PTAL3, TREPH2, ATEH2, and GREPH1 are labeled (Supplemental Table S5 d-f). *G* and *H,* bubble chart of gene ontology analysis performed on significantly regulated phosphorylated proteins following inversion (*G*) and gravistimulation (*H*). The analysis encompasses three categories: biological process, cellular component, and molecular function. The size of the dots represents the number of proteins associated with each enriched term, while the color indicates the base 10 logarithm ratio of the significance of each enriched term (Supplemental Table S4g, S5g). MS, mass spectrometry; TREPH, touch-regulated phosphoprotein; ATEH2, *Arabidopsis thaliana* EF-hand protein 2; PTM, posttranslational modification.

**Fig. 4:**
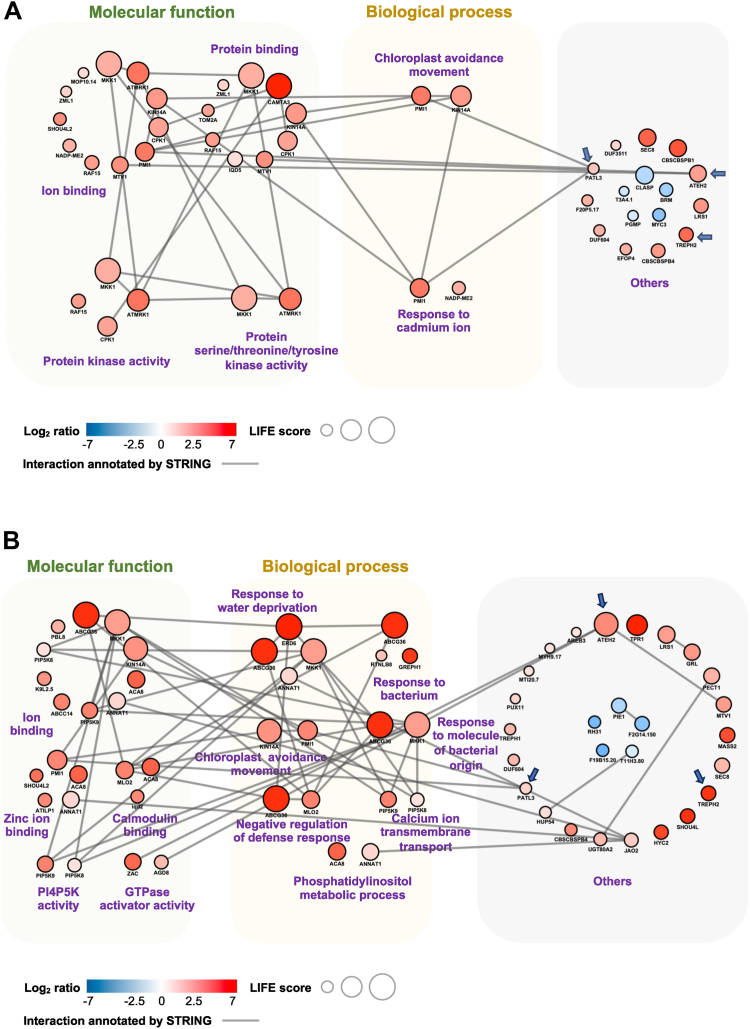
**Putative networks and clusters from gravity-regulated phosphoproteins.***A* and *B,* volvox graphic representations of the putative protein protein interaction from inversion (*A*), gravistimulation (*B*) results, respectively. *Circular nodes* represent significantly regulated phosphoproteins. The size of the node represents the likelihood of interaction and function evaluation (LIFE) score calculated by log2 ratio, GO analysis results, and STRING interaction score (Supplemental Tables S6 and S7). The *red and blue node* stands for the significantly upregulated and downregulated phosphoprotein, respectively. The color palette indicates the level of protein phosphorylation. The edge represents a probable protein–protein interaction predicted by the STRING database (Supplemental Tables S6 and S7). The thickness represents the confidence score (≥0.4) of interactions. The groups of phosphoproteins of the same function classified according to GO analysis are depicted into Volvox colony-like circles. The upper colonies on the *green background* were classified according to their molecular function. Colonies in the *yellow background* were classified according to their molecular function. The *blue arrow* marks the phosphoproteins, against which antibodies have been made (Supplemental Tables S8). GO, Gene Ontology.

**Fig. 5:**
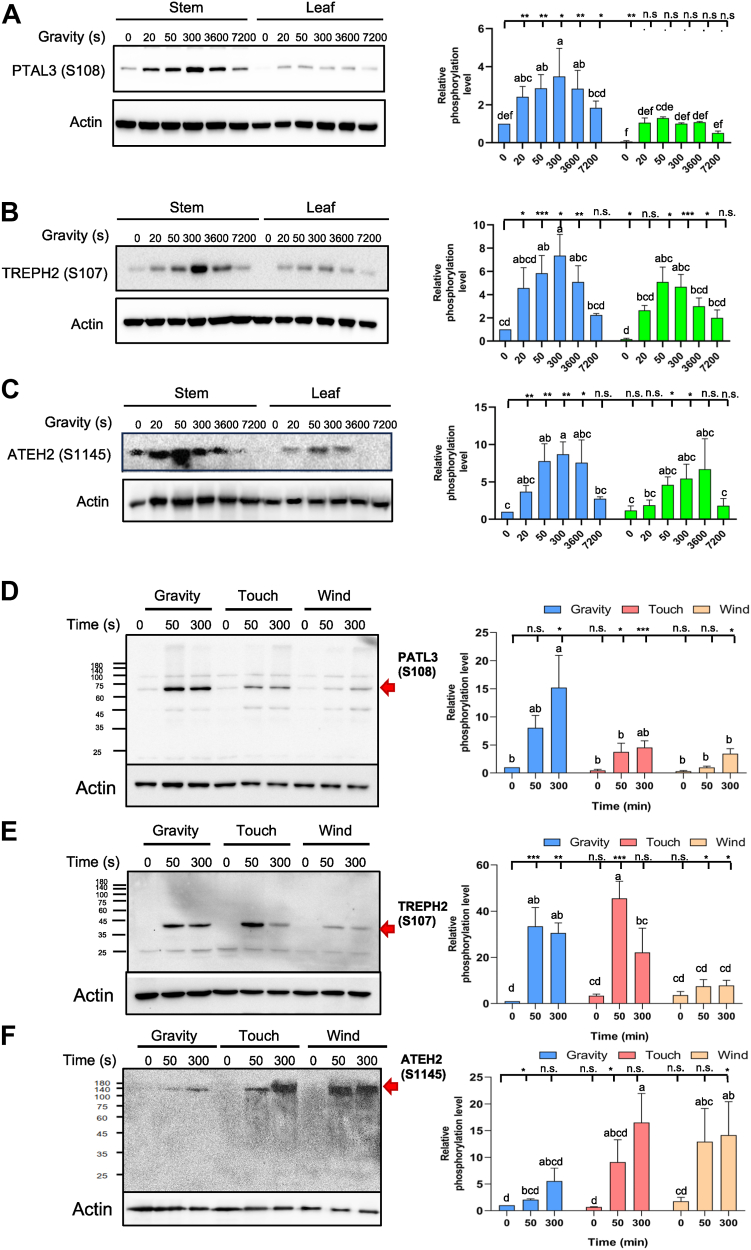
**Gravistimulation, touch, and wind stimulus-regulated protein phosphorylation in temporal and spatial fashion.***A–C,* immunoblotting measurement of the time course of phosphorylation level of phosphosite of PATL3-S108 (*A*), TREPH2-S107 (*B*), and ATEH2-S1145 (*C*) during 7200 s of gravistimulation (marked as gravity) in both stem and leaf of 25-day old Arabidopsis plants (Supplemental Figs. S9–S11). *D–F*, immunoblotting measurement of the time course of protein phosphorylation during 300 s of stimulation by gravity, touch, and wind in aerial organs of 25-day old plants for phosphosite of PATL3-S108 (*D*), TREPH2-S107 (*E*), and ATEH2-S1145 (*F*) (Supplemental Fig. S12). The relative levels of enhancement were measured using ImageJ and presented as bar graphs on the *right side* of immunoblots and data were collected from three biological replicates. The data were analyzed by Student’s *t* test; n.s. represents *p* > 0.05; ∗, ∗∗, and ∗∗∗ represents 0.01 < *p* < 0.05, 0.01 < *p* < 0.001, and *p* < 0.001, respectively. *Different letters* indicate significant differences at the 5% level shown above each bar, based on Tukey’s range test. *Red arrow* marks the targeted protein. PATL3, patellin 3; TREPH, touch-regulated phosphoprotein; ATEH2, *Arabidopsis thaliana* EF-hand protein 2.

**Fig. 6:**
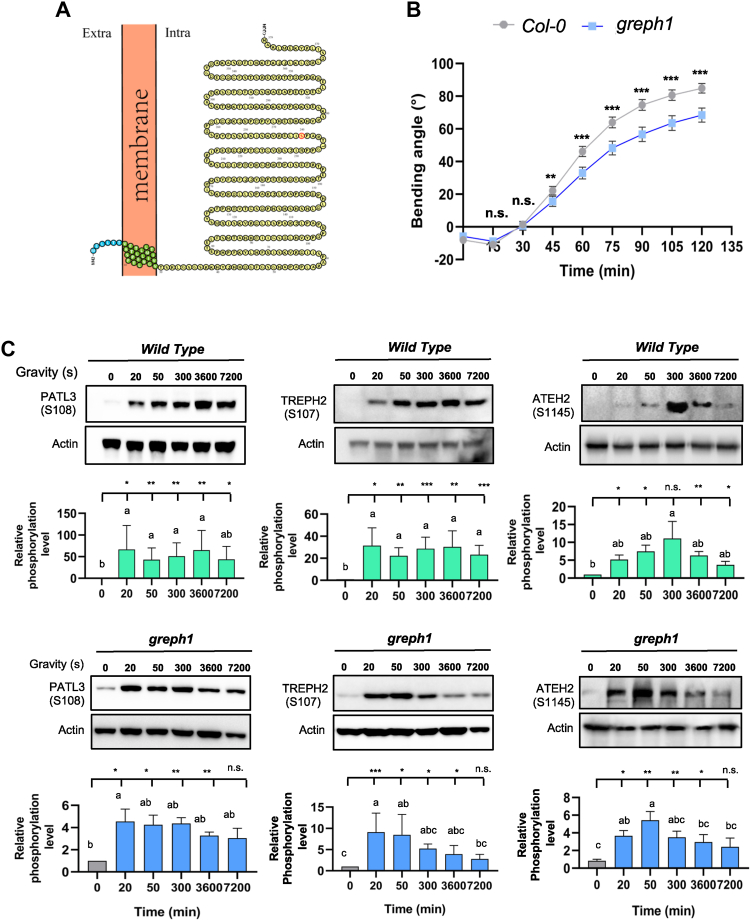
**The regulatory role of *GREPH1* in shoot gravicurvature and force-related protein phosphoprotein.***A,* the membrane topology of GREPH1 protein structure predicted by PROTTER online tool (82). The extracellular matrix domain, membrane domain, and cytosolic domain is marked in *blue, green,* and *yellow*, respectively. The phosphosite of GREPH1 protein is highlighted in *red*. *B,* the gravitropic response of the inflorescence stem of greph1 mutants and Col-0. Values are the average of at least 150 plants per genotype, with angles recorded every 15 min. Student's *t* test analyzed the greph1 mutant compared to Col-0 as reflected by *p* values (n.s. stands for *p* > 0.05; ∗∗∗ means *p* < 0.001) (Supplemental Figs. S13 and S14). *C,* immunoblotting measurement of the temporal changes of the phosphorylation level of phosphosite PATL3-S108, TREPH2-S107, and ATEH2-S1145, respectively, in 25-day old Arabidopsis inflorescence stem following one inversion of WT and greph1 mutant plants (See details from Experimental Procedures). The relative levels of enhancement were measured by ImageJ and presented as bar graphs of three biological replicates below the immunoblots in each figure. The data were analyzed by Student’s *t* test; n.s. represents *p* > 0.05; ∗, ∗∗, and ∗∗∗ represent 0.01 < *p* < 0.05, 0.01 < *p* < 0.001, and *p* < 0.001, respectively. *Different letters* indicate significant differences at the 5% level shown above each bar, based on Tukey's range test (three biological replicates are presented in the Supplemental Fig. S14, S15). TREPH, touch-regulated phosphoprotein; ATEH2, *Arabidopsis thaliana* EF-hand protein 2; PATL3, patellin 3.

To conduct the immunoblot analysis, the aerial plant organs, stems and leaves, of 25-day-old adult plants grown in Murashige and Skoog-based agar medium were collected by flash-freezing in liquid nitrogen, ground into fine powders, and extracted for the total cellular proteins using urea protein extraction buffer (UEB) buffer (51). The plants were inverted at a speed of six rpm and kept in an inverted state for different periods of time.

In the centrifugal force (inversion or rotation force) dose experiment, the 25-day-old adult plant were inverted for 1, 3, 7, and 9 times with a speed of 24 rpm, representing various dose of treatment. Plants treated with different doses of inversion stimulation were kept at downward position to 23 s in total before the liquid nitrogen harvest post the last inversion.

All inversion stimulation and gravistimulation experiments were performed under the horizontal green light irradiation conditions (Video 1–4).

The cotton touch experiment and wind stimulation were conducted according to previous published methods (52, 59).

Immunoblots were made by the following methods. Proteins of 25 μg were loaded onto either 10% or 12% SDS-PAGE gel. The SDS-PAGE gel fractionated proteins were transferred to polyvinylidene fluoride membranes (GE Healthcare). The membrane was incubated with both the primary and the secondary antibodies. Immobilon forte Western horse radish peroxidase) substrate (WBLUF0500, Millipore) was used for membrane exposure and signal detection. The secondary antibodies were anti-Mouse IgG-horse radish peroxidase (626520, Invitrogen) or anti-Rabbit IgG-peroxidase (A9169, Sigma) polyclonal antibodies, while the primary antibodies used were anti-actin mAb (a0480, Sigma Aldrich, 1:5000), anti-*p*S108-PATL3 polyclonal antibodies (GL Biochem, 1:1000) targeted to the sequence of MIPQNLG*p*SFKEESSC, anti-*p*S107-TREPH2 polyclonal antibodies (GL Biochem, 1:1000) targeted to the sequence of K*p*SINMPYFKFPQHNSEG, anti-*p*S167-TREPH2 polyclonal antibodies (GL Biochem, 1:1000) targeted to the sequence of GAPSLLQRVK*p*SIKL, and anti-*p*S1145-ATEH2 polyclonal antibodies (GL Biochem, 1:1000) targeted to the sequence of CYQRYD*p*SFNAQSYD. The specificity of these antibodies was tested against both phosphorylated and nonphosphorylated synthetic oligopeptides. The nonphosphorylated peptide containing the PTM site was used as a control.

### Protein Extraction

To perform *SILIA*-based quantitative phosphoproteomics on 20 s inversion and 30 s gravistimulation treated *Arabidopsis* organs, proteins were extracted separately either from the forward (F1, F2, F3) or reciprocal (R1, R2, R3) tissue powder mixture replicates (or called experimental replicates produced from three biological replicates, Supplemental Table S1). Proteins were extracted with four volumes (v/w = 4:1) of modified UEB containing 150 mM Tris (pH7.6), 8 M urea, 20 mM EDTA, 20 mM EGTA, 50 mM NaF, 1% G-2-P, 5 mM DTT, 1 mM PMSF, 0.5% phosphatase cocktail 2, Complete EDTA-free protease inhibitors cocktail, 5 mM ascorbic acid, and 2% PVPP (51, 55). The powder mixture was thoroughly mixed in a mortar pestle at room temperature. Protein extractions were centrifuged at 3000 rpm for 10 min at room temperature in a Sorvall ST8 mini benchtop centrifuge (Thermo Fisher Scientific) to remove cellular debris. CsCl salt was added into the protein extract to 2.8 M, which was centrifuged again at 28,000 rpm using SW28 rotor (Beckman) for 1.5 h at 12 °C to separate the cellular components into three fractions, including top, middle, and bottom. The proteins in the middle fraction (cytosol protein) were precipitated by three volumes (v/v) of precooled acetone/methanol buffer (v/v is 12:1) for at least 4 h in −20 °C, followed by 15 to 20 volumes (v/v) of acetone: methanol: H_2_O (v: v: v is 12:1:1.4) to wash the pellets. The precipitation and rinsing steps were repeated at least three times to ensure complete removal of the excess CsCl. After precipitation, the protein pellets were air-dried in a hood for about 30 min and then resuspended in the resuspension buffer (RSB) containing 6 M urea, 5 mM DTT, 0.3% sodium dodecanoate, 50 mM Tris–HCl (pH 8.0). This protein sample was defined as cytosolic protein. The top layer and bottom layer of CsCl gradient separation were combined and defined as membrane protein, which was further extracted using modified UEB consisting of 150 mM Tris (pH7.6), 8 M urea, 20 mM EGTA, 50 mM NaF, 1.2% Triton X-100, 1% SDS, 20 mM EDTA, 1% G-2-P and freshly added 0.5% phosphatase cocktail 2, Complete EDTA-free protease inhibitors cocktail, 5 mM DTT, 1 mM PMSF, and 5 mM ascorbic acid. The membrane protein was precipitated, rinsed according to the previous established procedure (56), and finally resuspended in RSB (Supplemental Table S1).

As to proteins used for the immunoblot analysis, the extraction method was quite different. First, 100 to 200 μl of tissue powders collected from the treated or control plant tissues were mixed with 450 μl UEB buffer for 10 min, centrifuged at 14,000 rpm for 10 min at 4 °C to remove cell debris, precipitated with precooled acetone: methanol (12:1, v:v), dried acetone, and dissolved with 8 M urea, 100 mM DTT, 0.5% SDS, and 50 mM Tris–HCl (pH 6.8). The concentration of the protein sample was measured using the DC protein assay analysis (Bio-Rad) and loaded on SDS-PAGE gel for fractionation and the immunoblot analysis.

### Alkylation and In-Solution Digestion

To reduce disulfide bonds, protein samples from both the membrane and cytosol were incubated with 5 mM DTT for 1 h at room temperature. The protein solution was treated with iodoacetamide to a final concentration of 50 mM, and the mixture was incubated in the dark at room temperature for 1 h to alkylate the cysteine residues in the proteins. After prewarming nine volumes of digestion buffer (consisting of 25 mM ammonium bicarbonate and trypsin at 1/20 the amount of protein), the RSB was slowly dripped into the buffer with continuous stirring at 37 °C. The proteins underwent two rounds of trypsin digestion. In the first round, they were digested at 37 °C in a shaker operating at 180 rpm for 12 to 16 h (trypsin: proteins are 1:20 w/w). For the second round, half the original amount (1:40 w/w) of fresh trypsin was added to the digestion buffer, and the digestion continued for an additional 6 h. The digested peptides were then acidified to a pH of 3 using formic acid (FA), causing the sodium dodecanoate to precipitate at the bottom of the solution. The precipitate was then removed by centrifugation at 28,000 rpm for 20 min at 14 °C using a SW 28 rotor, and the supernatant was subsequently filtered through a 0.22 μM cellulose acetate membrane filter (Sartorius). The peptides were desalted using a vacuum C18 column (Sep-Pak C18 35 cc Vac Cartridge, Waters) and washed with at least five volumes of 0.1% FA (v). The desalted peptides were eluted from the column using 80% acetonitrile and 0.1% FA. The acetonitrile was then evaporated from the solution using a fume hood with a blower to facilitate buffer exchange for the next experiment.

### Phosphopeptide Enrichment

The phosphopeptides were then enriched using established methods from prior studies (62). Phosphopeptides were enriched using a tandem purification method involving Titansphere TiO beads (GL Sciences) and Fe^3+^-chelated nitrilotriacetic acid (NTA) resin. First, peptides were dissolved in TiO_2_ loading buffer including 80% acetonitrile, 0.5% TFA, and 300 mg/ml lactic acid. The TiO_2_ beads were conditioned with a TiO_2_ wash buffer consisting of 80% acetonitrile and 0.5% TFA. This conditioning process was performed five times, with each round involving rotating the beads for 3 min and centrifuging them at 2000 rpm for 1 min using a Centrifuge 5418 R to remove the supernatant. Following the conditioning, the beads were equilibrated with the TiO_2_ loading buffer using the same process as the conditioning step, repeating this five times. The resuspended peptides were incubated with the equilibrated TiO_2_ beads for 45 min, followed by five washes with the TiO_2_ wash buffer. Phosphopeptides were eluted in 5% ammonium hydroxide (pH 12.0) and 5% pyrrolidine and then acidified with 0.1% TFA. The phosphopeptides underwent three rounds of TiO_2_ enrichment to enhance their purification. In the second round, the flow-through from the first round was further enriched for phosphopeptides. For the third round, the loading peptides were the eluates from the first and second enrichment steps.

The Fe^3+^-chelated NTA resin was used for immobilized metal affinity chromatography (IMAC)-based phosphopeptide enrichment. The NTA agarose (Qiagen) was first equilibrated with 6% acetic acid and then incubated with FeCl_3_ for 2 h in the dark at 4 °C with rotational mixing to prepare the Fe^3+^-chelated NTA resin. The excess FeCl_3_ was washed five times with 6% acetic acid and then equilibrated using an IMAC buffer containing 30% acetonitrile and 250 mM acetic acid. The peptides that passed through the second TiO_2_ enrichment were incubated with Fe^3+^-chelated NTA for 45 min, followed by five washes with IMAC buffer, each involving centrifugation for 1 min at room temperature. Phosphopeptides were then eluted using 5% ammonium hydroxide (pH 12.0), and the eluate was subsequently acidified with FA. The phosphopeptides were pooled from the three rounds of TiO_2_ enrichment and IMAC enrichment and then desalted and fractionated using strong cation exchange (SCX) and WAX columns.

### Phosphopeptide Fractionation

To minimize the impact of redundant peptides, we further separated the enriched phosphopeptides using ion exchange chromatography (63, 64). The dried peptide powder was resuspended in a solution containing 10 mM ammonium formate, 0.1% FA, and 20% acetonitrile. The phosphopeptides were separated using a BiopureSPN hydrophilicinteraction liquid chromatography-SCX column (The Nest Group), following the manufacturer's instructions. The column was activated and equilibrated, and then the peptides were loaded 5 times and centrifuged at 100 g for 1 min. The peptides were eluted using different percentages of SCX elution buffer, which consisted of 500 mM ammonium formate, 3% FA, and 20% acetonitrile, based on the amount of peptides present. The flow-through and wash buffer from the above process were then collected, mixed together, and subsequently desalted. The dried peptides were dissolved in a WAX loading buffer containing 5 mM Tris–HCl and 20% acetonitrile. The BioPureSPN HUM hydrophilicinteraction liquid chromatography-WAX column was equilibrated (The Nest Group), and then the loading and elution steps were the same as the SCX protocol, except that the SCX elution buffer was replaced by a WAX elution buffer consisting of 400 mM NaCl, 30% acetonitrile, and 0.05% FA.

### LC-MS/MS Analysis

The peptides were desalted using Ziptip peptide tips before being sent for LC-MS/MS analysis (Merk Millipore). The peptides were resuspended in solvent A (0.1% FA) and approximately 0.5 μg of the peptide was subsequently injected. The LC-MS/MS analysis of phosphopeptides was performed on an EASY-nLC 1200 UPLC system (Thermo Fisher Scientific) coupled with an Orbitrap Fusion Lumos Tribrid Mass Spectrometer (Thermo Fisher Scientific). An Easy-Spay RSLC C18 analytical column (Thermo) whose length was 15 cm and diameter was 75 μm was used to separate the peptides at a constant flow rate of 300 nl/min on a gradient of 0 to 10 min 2% solvent B, 10 to 13 min 2 to 5% solvent B, 13 to 85 min 5 to 30% solvent B, 85 to 87 min 30 to 50% solvent B, 87 to 89 min 50 to 5% solvent B, 89 to 94 min 5% solvent B, 94 to 95 min 5 to 2% solvent B, where solvent B was 0.1% FA in acetonitrile. The mass resolution was set at 120,000 for intact peptides (MS1) and at 30,000 for ion fragments (MS2) with an *m/z* range of 375 to 1500 under normalized collision energy of 30 activated by higher-energy collisional dissociation. The data-dependent acquisition mode was adopted for the intensity threshold which exceeded the ion count of 2 E^4^, and the exclusion duration was 30 s. A filtered charge state of 2 to 7 for a maximum injection time of 50 ms, an automatic gain control target value of 4 E^5^ charges, and the automatic gain control target value of MS/MS is 5 E^4^ whose maximum injection time is 100 ms. An isolation Window of 1.6 *m/z* was employed.

### Phosphopeptide Identification and Quantitation Using SILIA-Based SQUA, an In-House Software

The MS raw data which was generated from the Thermo spectrometer was converted into Mascot generic format and mzXML by MSCovert (version: 3.0.5084.0 64 bit) (57). The Mascot generic format files were searched and matched MS2 spectra on Mascot (version: version 2.6.0, 64 bit, Matrix Science) (64) based on TAIR10 (35,387 proteins, https://www.arabidopsis.org/download_files/Sequences/TAIR10_blastsets/TAIR10_pep_20101214_updated). The target-decoy strategy was used to estimate the false discovery rate (FDR). In this experiment, protein digestion was carried on by trypsin and the maximum of missed cleavage was set as 2. When the MS spectra matched with the database on Mascot, the setting was as follows: the mass tolerance was set as ± 10 ppm for MS1 and 0.02 Da for MS2. Carbamidomethyl (57.021464) on cystine (C) was set as a fixed modification. The variable modification contained phosphorylation (79.9663304 Da) on tyrosine (Y), serine (S), and threonine (T), and oxidation (15.994915 Da) on methionine (M). The quantitation method “^15^N metabolic” was specified. Mascot Percolator (Version 3.1) was appended to estimate the FDR. The peptide spectrum matching (PSM) cut-off threshold was set at 1%.

Quantification was carried out using an in-house software program called SQUA (58), which analyzed the DAT files generated from Mascot (64) and the mzXML files created through MSConvert (57). Phosphopeptides were chosen for quantification according to the following requirements: (i) the number of identified PSMs is larger than or equal to 2; (ii) the number of the identified light or heavy PSMs is larger than or equal to 1; (iii) the number of the ratios from different experimental replicates was larger than or equal to 3; (iv) the number of the ratios from the forward or reciprocal experiments divided by the total number of the ratios is larger than or equal to 0.2.

The selected quantifiable phosphopeptides were then calculated for their log_2_ ratio based on the maximum ion intensities of the paired light and heavy labeled ion chromatograms from the mass spectrometry analysis, using the aforementioned quantification criteria. Statistical significance was calculated through a two-tail student *t* test to determine if the mean of log ratios of a given unique PTM peptide array (UPA) significantly deviated from zero. All the binary logarithmic ratios were adjusted to be median-centered. The Benjamini–Hochberg (BH) method was then applied to correct for multiple hypothesis testing (BH), adjusting the q value derived from the *t* test and producing the BH-FDR. In this experiment, a BH-FDR threshold of 0.05 was used, indicating a 95% probability that the identified phosphopeptides were regulated by the treatment (either inversion stimulation or gravistimulation, Supplemental Tables S4–S7).

### Construction of Volvox Chart

Functional analysis, including gene ontology (GO) analysis and protein domain analysis, was performed using the DAVID bioinformatics resource (65, 66), with the InterPro method used for protein domain analysis (67). The figures of GO analysis including biological process (BP) enrichment, cellular component enrichment, and molecular function (MF) enrichment were made using the R with ggplot2 packages and GraphPad (https://www.graphpad.com/scientific-software/prism/). The significantly regulated phosphoproteins from the inversion and gravity stimulation experiments were submitted to the STRING database analysis (68), using a confidence score of 0.4 or higher, to identify putative connections between these phosphoproteins. Cytoscape (69) was used to visualize the Volvox network of proteins significantly regulated by inversion stimulation or gravity stimulation.

The likelihood of interaction and function evaluation (LIFE) score of a phosphoprotein (Supplementary Tables S6 and S7), which was significantly regulated by either inversion or gravistimulation treatment, was calculated by integrating GO analysis, STRING analysis, and the phosphorylation quantitation results according to previously published methods (59, 70). The score serves as a criterion used to select the phosphoprotein candidates for additional biological function validation.

## Results

### Experimental Design for Force-Loading–Specific Phosphoproteomics

Mechanical force perception in plants involves intricate signaling networks, with 180° inversion of Arabidopsis seedlings triggering biphasic cytoplasmic calcium transients (code 1 and 2) peaking at 4 s and 40 s (25), respectively. These distinct calcium signatures imply the existence of parallel signal transduction pathways for the centrifugal and gravitational forces generated during reorientation of plant organ. To dissect these calcium codes, we designed two complementary quantitative phosphoproteomic experiments on specifically treated *Arabidopsis* organs: (1) 20 s of multiple inversion (Inversion, covering the first stage of calcium spike response), probably capturing phosphorylation events (probably centrifugal force-responsive in theory because the actual forces involved in this type of inversion will be discussed in Discussion section) associated with calcium code 1 through acute 180° rotation at a speed of 24 rounds per minute (rpm, Fig. 1; Videos 1 and 2), and (2) 30 s upside-down hold (gravistimulation, covering the second stage of calcium spike response), presumably isolating calcium code 2–specific phosphoproteomic response by incorporating both a 30 s upside-down hold and an initial 20 s repetitive inversion (Fig. 1; Videos 3 and 4), subsequently subtracting phosphorylation events from 20 s multiple inversions by phosphoproteomic quantitation, leading to identification of 30 s gravistimulation-specific phosphoprotein groups.

Each gravity force treatment-specific experiment included six experimental replicates of tissue mixtures, with approximately 60 g of mixed liquid nitrogen frozen powders of aerial tissues collected per biological replicate. The six tissue mixtures (F1, F2, F3, R1, R2, and R3; Supplemental Table S1) were generated by reciprocal mixing of tissue samples of three biological replicates. Given the proposed role of speculative membrane-bound kinase receptors and ion channels in force signaling, we employed a subcellular fractionation method, CsCl density gradient centrifugation, to separate cytoplasmic and membranous proteins (58, 63), enabling enrichment of membrane-bound phosphoproteins and targeting LC-MS/MS analysis of phosphopeptides from 12 subcellular fractions of proteins from those six tissue mixtures (Supplemental Figs. S1–S3).

### Identification of Phosphopeptides and Phosphoproteins

A total of 24 cytosolic and membrane protein samples (12 subcellular fractions × two force treatments = 24 protein fraction samples) were isolated from the *Arabidopsis* aerial organs subjected either to inversion or gravistimulation (Fig. 1) via CsCl density gradients (Supplemental Fig. S1). Following tryptic digestion and phosphopeptide enrichment, more than 180 phosphopeptide fractions (90 fractions per force treatment) were analyzed by LC-MS/MS. Using Mascot (1% FDR), we identified 127,104 (inversion) and 132,338 (gravistimulation) redundant phosphopeptides, with 6625 and 7328 unique, reproducible phosphopeptides (PSMs ≥2), respectively (Fig. 2, *A* and *B*; Supplemental Fig. S2*A*; Supplemental Tables S2 and S3). Phosphopeptide discovery rates increased modestly (0.5–3%) beyond the fourth replicate (Supplemental Fig. S2*B*; Supplemental Tables S2 and S3). Phosphopeptide abundance followed Zipf's law (slopes: −0.58 inversion, −0.57 gravistimulation; Supplemental Fig. S2*C*; Supplemental Tables S2 and S3), suggesting a similar degree of penetration by phosphoproteomic analysis between the two experiments. The more of the lower abundance proteins MS/MS identifies, the larger the negative slope of Zipf’s law is. Gravistimulation phosphopeptides exhibited higher phosphorylation site multiplicity (848 doubly, 100 triply, six tetraphosphorylated, 1 pentaphosphorylated) *versus* inversion (550, 33, 2, 0; Supplemental Fig. S3*A*; Supplemental Tables S2 and S3). Most phosphopeptides were 8 to 22 aa long (76% inversion, 78% gravistimulation; Supplemental Figs. S3*B*; Supplemental Tables S2 and S3). These mapped to 2733 (inversion) and 2878 (gravistimulation) phosphoproteins (Fig. 2*F*; Supplemental Figs. S2 and S3), predominantly 30 to 110 kDa (81%, 86%; Supplemental Fig. S3*C*; Supplemental Tables S2 and S3). We identified 5215 (inversion) and 5712 (gravistimulation) phosphosites, with 1152 and 1283 novel sites versus PhosPhAt 4.0 (Supplemental Fig. S3*D*; Supplemental Tables S2 and S3). Motif analysis showed Inversion: 35% acidophilic (1,819), 15.8% basophilic (820), 35.1% proline-directed (1,824); gravistimulation: 31.2% (1779), 17% (969), 37.2% (2121) (Fig. 2, *C* and *D*; cf. PhosPhAt 4.0: 17.4% proline-directed; Fig. 2*E*). Shared phosphoproteins totalled 2,337, with 396 inversion-associated and 541 gravistimulation-associated (Fig. 2*F*; Supplementals Tables S2 and S3). Novel phosphoproteins included 201 (inversion) and 218 (gravistimulation) (Supplemental Fig. S4; Supplemental Tables S2 and S3). Differential phoshoproteomic comparisons revealed 126 novel phosphoproteins common to both experiments, plus 75 (inversion) and 92 (gravistimulation) unique (Fig. 2*G*). Most phosphoproteins had ≤5 sites; only 3.6% (98/2735) and 4.6% (134/2885) possessed ≥6 sites (Fig. 2*H*). High-site examples included 19 sites for IQ domain protein 14 (containing 25 aa long polypeptide with motifs [I, L, V]QxxxRGxxx[R,K]) and 28 sites for serine/arginine (R) repetitive matrix protein 1-little protein in the inversion and gravistimulation experiment, respectively. Phosphoprotein distribution followed exponential decay relative to site number (Fig. 2*H*; Supplemental Fig. S5*A*; Supplemental Tables S2 and S3). No correlation existed between phosphosite number and S/T/Y residues, protein length, or molecular weight (Pearson r = 0.18–0.22; Supplemental Fig. S5, *B*–*D*; Supplemental Tables S2 and S3), suggesting a specific regulation of protein phosphorylation in plant cells upon treatments rather than random phosphorylation of polypeptides by kinases.

### Quantitative and Functional Phosphoproteomics Decodes the Cellular Functions of Force Treatment-Specific Phosphoproteins

Quantitative phosphoproteomic analysis employing UPAs and UPSPs (52, 53, 54, 56); revealed distinct phosphorylation landscapes under mechanical force stimulation. Inversion treatment generated 5585 UPAs (Supplemental Table S4), with 4708 UPAs containing single phosphopeptides and 23 containing ≥3 phosphopeptides (Supplemental Fig. S6*A*). Gravistimulation yielded 6145 UPAs (Supplemental Table S5), including 5147 single-phosphopeptide UPAs and 21 with ≥3 phosphopeptides (Supplemental Fig. S6*C*). The 10% increase (560 UPAs) under gravistimulation indicates gravity-specific phosphorylation amplification. Both datasets followed power-law distributions (inversion slope: |0.58–0.63|; gravistimulation: |0.57–0.59|; Supplemental Fig. S6, *B* and *D*). Subcellular fractionation via CsCl density gradients fractionation (Supplemental Fig. S1*A*) enabled compartment-specific quantification using SQUA (58, 64). For inversion, cytosolic fractions showed 14 upregulated and three downregulated unique phosphopeptides (UPSPs; FDR ≤0.05, |FC|≥1.5; Fig. 3*A*; Supplemental Table S4), while membrane fractions exhibited 14 upregulated UPSPs (Fig. 3*B*; Supplemental Table S4). Combined analysis identified 29 upregulated and five downregulated UPSPs, 34 inversion treatment significantly regulated in total (Fig. 3*C*; Table 1; Supplemental Table S4). In gravistimulation, cytosolic fractions contained 26 upregulated and six downregulated UPSPs (Fig. 3*D*; Supplemental Table S5), membrane fractions showed 24 upregulated and six downregulated UPSPs (Fig. 3*E*; Supplemental Table S5), and total fractions revealed 47 upregulated and five downregulated gravity force–specific UPSPs, 52 gravistimulation treatment significantly regulated in total (Fig. 3*F*; Table 1; Supplemental Table S5). Comparative analysis identified 16 commonly shared UPSPs responsive to both stimuli (Table 1), indicating shared phospho-relay mechanisms, and discovered a novel phosphosite on ADP-ribosylation factor GTPase-activating protein 12, a regulator of EHB1 distribution during gravitropism (71), suggesting that there exist gravity stimulus-specific signaling components in the commonly shared phosphoprotein cohort.

**Table 1:** Inversion and gravistimulation significantly regulated phosphosites (UPSP)^a^

No.	Protein group-UPSP^b^	Protein annotation^c^	Fold change^d^
1	AT1G80490.1-*p*T285; AT1G80490.2-*p*T286	TPR1 | topless-related 1	641.1
2	AT1G08930.1-*p*S6-oxM7; AT1G08930.2-*p*S6-oxM7	*ERD6 | major facilitator superfamily protein*	474.6
3	AT1G59870.1-*p*S825	*ABCG36 | ABC-2 and plant PDR ABC-type transporter family protein*	26.4
4	AT1G16860.1-oxM94-*p*S105-oxM106	SHOU4L | ubiquitin-specific protease family C19-related protein	25.2
5	AT5G56980.1-*p*S240	***GREPH1 | gravity-regulated phosphoprotein 1***	**22.4**
6	AT5G64090.1-*p*S430	HYC2 | part of a nanodomain complex that tethers PI4Kα1 to the plasma membrane	18.5
7	AT1G15400.1-pS10; AT1G15400.2-pS10; AT1G15400.3-pS10; AT1G80180.1-pS10	MASS1/2 | MAPK substrates in the stomatal lineage 1/2	16.5
8	AT5G57110.1-pS57; AT5G57110.2-pS57	*ACA8 | autoinhibited Ca2+ -ATPase, isoform 8*	11.6
9	AT4G21160.1-pS155; AT4G21160.2-pS155; AT4G21160.3-pS155; AT4G21160.4-pS155	ZAC | calcium-dependent ARF-type GTPase activating protein family	10.8
10	AT1G11310.1-*p*S570	*MLO2 | seven transmembrane MLO family protein*	7.2
11	AT3G09920.1-pS11; AT3G09920.2-pS11; AT3G09920.3-pS11	PIP5K9 | phosphatidyl inositol monophosphate 5 kinase	7.1
12	AT3G62700.1-*p*S894	ABCC14 | multidrug resistance-associated protein 10	7
13	AT1G42550.1-*p*S415-oxM418	PMI1 | plastid movement impaired1	6.7
14	AT1G17210.1-*p*S302	ATILP1 | IAP-like protein 1	6.4
15	AT5G03040.1-pS296; AT5G03040.2-pS296; AT5G03040.3-pS296	iqd2 | IQ-domain 2	6.4
16	AT5G44290.1-pT583-oxM584; AT5G44290.2-pT583-oxM584; AT5G44290.3-pT583-oxM584; AT5G44290.4-pT583-oxM584	K9L2.5 | protein kinase superfamily protein	5.3
17	AT2G35110.1-pS789; AT2G35110.2-pS760	GRL | transcription activators	5
18	AT3G05090.1-pS386; AT3G05090.2-pS386	LRS1 | transducin/WD40 repeat-like superfamily protein	2.2
19	AT5G01020.1-*p*S31	PBL8 | protein kinase superfamily protein	3.4
20	AT2G38670.1-*p*S416	PECT1 | phosphorylethanolamine cytidylyltransferase 1	3.4
21	AT5G55860.1-*p*S625	*TREPH1 (WPRa4) | touch-regulated phosphoprotein 1*	3
22	AT3G07020.1-pT82; AT3G07020.2-pT82	UGT80A2 | UDP-glycosyltransferase superfamily protein	3
23	AT4G17890.1-*p*S402	AGD8 | ARF-GAP domain 8	2.9
	AT3G10260.1-pS238; AT3G10260.2-pS238; AT3G10260.3-pS258	RTNLB8 | reticulon family protein	2.6
24	AT2G43210.1-pS280; AT2G43210.2-pS280	PUX11 | ubiquitin-like superfamily protein	2.9
25	AT1G35720.1-*p*S14	ANNAT1 | annexin 1	1.9
26	AT4G27450.1-*p*S238	HUP54 | aluminium-induced protein with YGL and LRDR motifs	1.7
27	AT5G09960.1-*p*S54	MYH9.17	1.6
28	AT1G60890.1-oxM609-pS610; AT1G60890.2-oxM621-pS622	PIP5K8 | phosphatidylinositol-4-phosphate 5-kinase family protein	1.6
29	AT5G57830.1-*p*S154	MTI20.7	1.6
30	AT3G56850.1-*p*S43-oxM44	AREB3 | ABA-responsive element-binding protein 3	1.6
31	AT5G25070.1-*p*S669	T11H3.80	0.6
32	AT3G12810.1-*p*T1578	PIE1 | SNF2 domain-containing protein/helicase domain-containing protein	0.3
33	AT5G15030.1-pS171; AT5G15030.2-pS207	F2G14.150 | paired amphipathic helix (PAH2) superfamily protein	0.2
34	AT5G63630.1-*p*S175	RH31 | P-loop–containing nucleoside triphosphate hydrolases superfamily protein	0.2
36	AT4G28990.1-pS275; AT4G28990.2-pS323	F19B15.20 | RNA-binding protein-related	0.1
37	AT4G26130.1-*p*S107-oxM110	*TREPH2 | touch-regulated phosphoprotein 2*	**24.4**
38	AT4G26130.1-*p*S167	***TREPH2 | touch-regulated phosphoprotein 2***	**5**
39	AT4G22290.1-*p*S150	SHOU4L2 | ubiquitin-specific protease family C19-related protein	9.5
40	AT1G42550.1-*p*S41	PMI1 | plastid movement impaired1	4.2
41	AT1G21630.1-pS1172; AT1G21630.2-pS1201	*ATEH2 | Arabidopsis Thaliana EH domain-containing protein 2*	**6.4**
42	AT5G50530.1-pS18; AT5G50640.1-pS18	CBSCBSPB4 | CBS/octicosapeptide/Phox/Bemp1 (PB1) domains-containing protein	6.3
43	AT5G10470.1-pS46; AT5G10470.2-pS46	KAC1 (Kin14A) | kinesin-like protein for actin-based chloroplast movement 1	5.5
44	AT4G26070.1-pT29; AT4G26070.2-pT29; AT4G26070.3-pT29; AT4G29810.1-pT31	*MKK1/2| MAP kinase kinase 1/2*	4.7
45	AT3G16270.1-*p*T211	MTV1 | ENTH/VHS family protein	4.7
46	AT3G16270.1-*p*T211-oxM212	MTV1 | ENTH/VHS family protein	2.9
47	AT3G05090.1-pS379-pS386; AT3G05090.2-pS379-pS386	LRS1 | transducin/WD40 repeat-like superfamily protein 1	4.6
48	AT4G15240.1-*p*S19-oxM24	DUF604 | protein of unknown function (DUF604)	3
49	AT3G10380.1-*p*T250	SEC8 | subunit of exocyst complex 8	3
50	AT5G05600.1-*p*S29	JAO2 | 2-oxoglutarate (2OG) and Fe(II)-dependent oxygenase superfamily protein	2.2
51	AT1G72160.1-oxM101-*p*S108	*PATL3 | Sec14p-like phosphatidylinositol transfer family protein*	**2**
52	AT1G72160.1-*p*S108	***PATL3 | Sec14p-like phosphatidylinositol transfer family protein***	**2**
53	AT2G22300.1-*p*S964;AT2G22300.2-*p*S964;AT5G09410.1-*p*S924;AT5G09410.2-*p*S942;AT5G09410.3-*p*S1001	SR1 | signal responsive 1; CAMTA| calmodulin-binding transcription activator	94.4
54	AT5G63490.1-*p*S12	CBSCBSPB1| CBS/octicosapeptide/Phox/Bemp1 (PB1) domains-containing protein	14.6
55	AT3G63260.1-*p*T41;AT3G63260.2-*p*T41	*ATMRK1 | protein kinase superfamily protein*	8.9
56	AT3G58640.1-*p*S371;AT3G58640.2-*p*S371	RAF15 | mitogen-activated protein kinase kinase kinase-related	4.6
57	AT1G21630.1-*p*S1145;AT1G21630.2-pS1174	***ATEH2 | calcium-binding EF-hand family protein***	4.5
58	AT5G04870.1-*p*S605	CPK1 | calcium-dependent protein kinase 1	4.3
59	AT1G32400.1-*p*S260;AT1G32400.2-*p*S260;AT1G32400.3-*p*S260	TOM2A | tobamovirus multiplication 2A	4.2
60	AT5G26850.1-*p*S594;AT5G26850.2-*p*S594;AT5G26850.3-*p*S594;AT5G26850.4-*p*S594	EFOP4 | uncharacterized protein	3.4
61	AT5G11670.1-oxM579-*p*S581	NADP-ME2 | NADP-malic enzyme 2	3.35
62	AT1G70100.1-*p*S99;AT1G70100.2-*p*S99;AT1G70100.3-*p*S99;AT1G70100.4-*p*S99	F20P5.17 | unknown protein	3.3
63	AT3G21175.1-pS121;AT3G21175.2-pS121;AT3G21175.3-pS1210	ZML1 | ZIM-like 1	2
64	AT3G13910.1-*p*S60	DUF3511 | protein of unknown function (DUF3511)	1.9
65	AT3G22190.1-pS42;AT3G22190.2-*p*S42	IQD5 | IQ-domain 5	1.7
66	AT5G51820.1-oxM178-*p*S181	PGM | phosphoglucomutase	0.6
67	AT2G46630.1-*p*S102-*p*T105-*p*T111	T3A4.1 | unknown protein	0.5
68	AT2G20190.1-*p*S1051	CLASP | CLIP-associated protein	0.4
69	AT2G46020.1-*p*S1884;AT2G46020.2-*p*S1885	CHR2 | transcription regulatory protein SNF2, putative	0.3
70	AT5G46760.1-pS376-*p*S379	MYC3 | basic helix-loop-helix (bHLH) DNA-binding family protein	0.2

Bold protein names represent those that have been studied using immunoblots and/or genetics.

Italicized marks those genes being knocked out by CRISPR method and having been used for gravitropic mutant screen.

Underlined are overlapping phosphoproteins and UPSPs found from both gravistimulation (No. 1–36)-specific phosphoproteins and inversion-specific phosphoproteins (No. 53–70).

ACA8, calcium-transporting ATPase 8; IQD2, IQxxxRGxxxR domain-containing nuclear protein 2; PTM, posttranslational modification; BH, Benjamini–Hochberg; FDR, false discovery rate.

a Criteria used to select the significantly regulated unique PTM site patterns (UPSPs): 1. BH-FDR ≤0.05; 2.| Log ratio| ≥ 0.6, |Fold change| ≥ 1.5; 3. Biological replicates = 3; 4. Experimental replicates ≥4.

b Phosphopeptide sequences share the same UPSP, and p represents the phosphoylated amino acid.

c Protein abbreviation and annotation of the leading protein.

d Fold change of protein phosphorylation level either by gravistimulation or inversion.

To study the functions of force treatment significantly regulated phosphoproteins, GO enrichment analysis of phosphoprotein groups containing significantly regulated unique phosphosites (Fig. 3, *G* and *H*; Table 1) revealed distinct biological relevance for each stimulus. Inversion-regulated phosphoproteins were uniquely enriched in BPs response to cadmium and zinc ions, whereas gravistimulation-regulated phosphoproteins were differentially enriched in actin nucleation, nucleosome assembly, response to bacterium and water deprivation (Fig. 3). Cellular component analysis showed gravistimulation-specific phosphoproteins exhibited a broader enrichment, especially suppressor of cAMP receptor complex. According to molecular function classification, only the gravistimulation-specific phosphoproteins showed a unique enrichment in 1-phosphatidylinositol-4-phosphate 5-kinase (PI4P5 kinase) activity and calmodulin-binding, which are known gravitropism regulators (72); Fig. 3), as well as a P-type calcium transporter activity.

Motif-X analysis of upregulated phosphorylation UPSPs identified a conserved LxRxxSI motif in both inversion and gravistimulation experiments (Supplemental Fig. S6, *E* and *F*). Gravistimulation additionally revealed a conserved QRxxSI/F motif.

InterPro domain analysis further differentiated treatment-specific phosphoproteins. Inversion-specific proteins were enriched in six domains, including three kinase domains (Ser/Thr kinase AS, protein kinase domain, kinase-like domain; Supplemental Fig. S6*G*). Gravistimulation-specific proteins showed enrichment in 11 domains, notably PI4P5 kinase and ADP-ribosylation factor GTPase-activating protein (ARF-GAP) domains (Supplemental Fig. S6*H*).

### Volvox Chart Maps the Functional Modules of Force Treatment-specific Phosphoproteins

To show the overarching functions of force treatment-specific phosphoproteins in signaling networks, the functional clusters were generated using the LIFE score and Volvox chart (Fig. 4; Supplemental Tables S6 and S7). The LIFE score was calculated based on phosphorylation level, GO and STRING analysis results of treatment-specific phosphoproteins (See Experimental Procedures for details). It was found that the inversion-specific phosphoproteins formed two BP clusters (chloroplast avoidance movement, response to cadmium ion) and four MF clusters (ion binding, protein binding, protein kinase activity, protein serine/threonine/tyrosine kinase activity) (Fig. 4*A*, Supplemental Table S6). These inversion force probably triggered calcium spike 1–speicifc proteins are either calcium sensors (EF-hand protein), mitogen-activated calcium signal transducer, Calcium-dependent Protein Kinase 1 (CPK1) and calmodulin-interacting proteins or proteins in Mitogen-Activated Protein Kinase (MAPK) cascades including *Arabidopsis thaliana* MLK/Raf-related protein kinase 1 and Rapidly Accelerated Fibrosarcoma 15 (RAF15), suggesting that the chloroplast-triggered cellular tension, chloroplast sedimentation (or chloroplast movement) and kinase activity are the initial physical and biochemical signals generated in response to reorientation (rotation) of plant organs. Conversely, gravistimulation-specific phosphoproteins generated seven BP clusters of proteins having response to bacterium, response to water deprivation, chloroplast avoidance movement, response to molecule of bacterial origin, negative regulation of defense response, calcium transmembrane transport, phosphatidylinositol metabolic process and 5 MF clusters of proteins having ion binding, zinc binding, calmodulin binding, PI4P5 kinase activity, and GTPase activator activity (Fig. 4*B*, Supplemental Table S7). These phosphoproteins can be further divided into the overarching subfunction groups of calcium signaling and homeostasis proteins including calcium-transporting ATPase 8, zinc-finger/ARF-GAP/C2 (ZAC) as in ARF-GAP protein 12 (ADP-ribosylation factor GTPase-activating protein), calmodulin-binding and IQxxxRGxxxR (IQ) Domain-containing nuclear protein 2 (IQD2), Annexin 1 (ANNAT1), central to force signaling pathway, membrane vesicle trafficking protein, ABC transporter G family member 36 (ABCG36) and C family member 14 (ABCC14), ARF-GAP 8 (AGD8), Reticulon-like protein B8 (RTNLB8) and lipid signaling proteins, phosphatidylinositol 4-phosphate 5-kinase and phosphorylethanolamine cytidyltransferase 1, supporting stress signaling molecule transport and finally hormone/stress signal transduction proteins, Topless-related 1, ABA responsive element binding protein 3, TREPH1, hypoxia response unknown protein 54, mildew resistance locus O-like protein 2, transcription activator (GRL) orchestrating responses to environmental cues. Taken together, these phosphoproteins found from the two stages (1–20 s and 20–50 s) of organ reorientation time course have distinct MFs in both cellular and BPs in response to both centrifugal and gravitational mixed forces (20 s of inversions) and that generated gravitational force alone (30 s).

### Stem-Enriched Phosphosignatures Drive Gravity Force–specific Signaling in *Arabidopsis* Gravitropism

*SILIA*-based quantitative phosphoproteomics identified 34 inversion-specific phosphopeptides (or UPSPs) and 52 gravistimulation-specific phosphopeptides, with 16 phosphosites conserved across both stimulations (Table 1). According to previously reported results, the phosphorylation of phosphoproteins, PATL3 (pS108) and TREPH2 (pS107 and p167), was responsive to gravity force and touch force, respectively (52, 54). However, in these phosphoproteomics experiments, it was found that the phosphorylation of phosphosites, *p*S108-PATL3, *p*S107-TREPH2, and *p*S167-TREPH2, were found from plants under both Inversion and gravistimulation treatments while the phosphorylation of *p*S1145-ATEH2 responded only to the inversion treatment specifically (Table 1). Antibodies made against all three phosphoproteins showed no cross-reactivity in the loss-of-function *patl3*, *treph2*, and *ateh2* mutants (Supplemental Fig. S8). Immunoblot analysis confirmed the quick phosphorylation enhancement for all four phosphosites following one time of inversion (6 rpm) and hold upside-down for either 20 s, 50 s, or 5 min (300 s) (Supplemental Figs. S9 and S10), validating the quantitative phosphoproteomics results (Fig. 3).

Inversion dose assay started from a single inversion to nine consecutive inversions (Supplemental Fig. S9). A single inversion treatment (with a rotation speed of 24 rpm) and the repeated inversion treatments (2–9 times) uniformly upregulated phosphorylation without additive effects (See Experimental Procedures for details; Supplemental Fig. S9), supporting the implementation of single-inversion protocol for gravity response isolation in the following immunoblot experiment. *AEQ* transgenic plants exhibited identical phosphorylation kinetics to the WT *Col-0* under the gravistimulation treatment (Supplemental Fig. S10), confirming conserved phosphor–relay pathways, which is unaffected by the existence or *AEQ* transgene in plant cells.

Temporal analysis of the inflorescence stem revealed both a phosphosite-specific rapid increase in phosphorylation and phosphor-decay patterns for numerous phosphosites examined in both stem and leaf tissues (Fig. 5; Supplemental Figs. S10–S12). Spatiotemporal dissection demonstrated the stem-specific phosphorylation enhancement peaking at 5 min (300 s) poststimulation for *p*S108-PATL3, *p*S107-TREPH2, and *p*S1145-ATEH phosphosites, while their phosphorylation levels gradually decayed by 2 h (7200 s) (Fig. 5, *A*–*C*). In leaves, their phosphorylation levels exhibited similar up-and-down variations, except at a relatively lower range of phosphorylation level (Fig. 5, *A*–*C*).

Comparative force stimulus profiling revealed distinct phosphosite responses to various mechanical forces (Fig. 5, *D*–*F*). The *p*S108-PATL3 showed maximal gravity force induction (15-fold increase at 300 s) *versus* touch force (4.5-fold) or wind force (3.5-fold) responses. The *p*S107-TREPH2 exhibited lower gravity-specific amplification (30-fold increase at 300 s) than that of touch (45-fold at 50 s decreasing to 22-fold at 300 s) but higher than that of wind (eight-fold) responses. The *p*S1145-ATEH2 demonstrated weaker gravity response (five-fold increase at 300 s) than touch (16-fold) or wind (14-fold) stimulation (Fig. 6). Taken together, phosphorylation on *p*S108-PATL3 is more responsive to gravity signal than touch force and wind force signals, whereas *p*S107-TREPH2 and *p*S1145-ATEH2 are relatively touch and wind force-responsive. These results are consistent with the previously reported (52, 54) as well as phosphoproteomics results from this study (Table 1).

### GREPH1 Plays a Role in Regulation of Forces-Induced Phosphorylation and Shoot Gravitropism

To validate their biological roles of phosphoproteins in gravitropism, candidates from inversion- and gravistimulation-specific and commonly shared phosphoproteins (Table 1) were selected based on phosphorylation levels, LIFE scores (Supplemental Tables S6 and S7), and functional Cluster analysis with LIFE score calculation (Fig. 4) to generate many gene knockout mutants (Supplemental Fig. S13). Together with several prior phosphoproteomic hits (52, 54, 59), a total of 17 CRISPR/Cas9 single/double mutants were generated in the mutagenesis experiments for a mutant screen for shoot gravitropism defects (Supplemental Fig. S13). The gravitropism mutant screening of the light-grown seedlings revealed that *greph1* exhibited severely impaired gravicurvature (<15°/day *versus* >42°/day in the WT). Mutants *mkk1*, *erd6mol2*, and *erd6aca8* showed moderate impairment (<30°/day), while *patl3*, *ateh2*, and *treph2* mutants exhibited WT-like gravitropic responses unexpectedly in these functional assays (repeated twice; Supplemental Fig. S13).

We therefore focused on the integrin-like protein GREPH1 (Fig. 6*A*) to study its role in the gravitropism of inflorescence stem using *greph1* mutant lines (Supplemental Fig. S13). Following gravity vector change, *greph1* inflorescence stems showed a significantly reduced gravicurvature (68.5 ± 2.17°) *versus* *Col-0* (84.86 ± 1.49°) and *AEQ* controls (83.4 ± 2.02°) at 2 h (7200 s) (Fig. 6*B*, Supplemental Fig. S14).

All three phosphosites, *p*S108-PATL3, *p*S107-TREPH2, and *p*S1145-ATEH2, which were significantly upregulated by 20s force treatments, showed a rapid hyperphosphorylation at 20 s poststimulation (Fig. 6*C*; Supplemental Fig. S11). Phosphorylation kinetics of *p*S108-PATL3 is similar in both *greph1* mutant and the WT (Fig. 6*C*; Supplemental Fig. S15). The phosphorylation of this site increased at 20 s poststimulation, consequently stayed the same for the rest of 1 to 2 h (3600 s–7200 s), as compared to its phosphorylation in the WT, which stayed the same for 1 h (3600 s) and slightly reduced by 2 h (7200 s) (Fig. 6*C*). As to *p*S107-TREPH2 phosphosite, the phosphorylation stayed unchanged over 2 h (7200 s) in the WT plants while, in the *greph1* mutant, the phosphorylation of this site peaked between 20 s and 50 s and gradually decayed by 2 h (7200 s) (Fig. 6*C*). Moreover, the phosphorylation of pS1145-ATEH2 phosphosite peaked at 20 s to 50 s in *greph1* mutant, whereas its upregulated phosphorylation lasted for 2 h in the WT (Fig. 6*C*). Especially, for pS1145-ATEH2 site which is specific to 20 s inversion treatment, the *greph1* mutation shifted its phosphorylation peak to 50 s as compared to that of the less likely altered phosphorylation for 2 h (7200 s) postinduction in the WT. In summary, the defective *greph1* gene triggers a quick phospho-decay post the phosphorylation peaks occurring either at 20 s or 50 s for *p*S107-TREPH2 and *p*S1145-ATEH2 site (Fig. 6*C*; Supplemental Fig. S15). These influential effects of *greph1* mutant gene over these force stimulus-induced phosphorylation suggest a key role played by *GREPH1* in mediating the force signaling (Fig. 6; Supplemental Fig. S15).

## Discussion

Rapid signal-induced phosphorylation dynamics constitute a “PTM code,” while stimulus-triggered calcium spikes represent a “calcium code.” In plant organ reorientation, a 180° rotation, generates an initial calcium transient at 4 s (calcium code 1) and a subsequent spike at 40 s (calcium code 2). Deciphering how these calcium codes interface with phosphorylation-mediated signaling (PTM code) is a pivotal biological question. Yet, the causal relationship between rapid PTM responses and calcium transients remains unexplored. Here, we employed *SILIA*-based quantitative phosphoproteomics to identify immediate, phosphorylation changes (PTM Codes) linked to each calcium code (Fig. 1).

A single organ inversion simultaneously generates both centrifugal and gravitational forces in theory. In our inversion experiments (Fig. 1; Videos 1 and 2), we increased rotational speed to 24 rpm (*versus* previously used 6 rpm) to amplify centrifugal force, assuming gravitational variation was negligible. However, the rotation of a whole plant also introduced flexing or bending of plant organs, such as inflorescence stem, at the end of inversion, which has been considered as a distinct type of force loading. As such, 34 unique phosphopeptides detected from inversion experiment (Table 1) are supposed to be responsive to three types of forces: centrifugal force, gravity force, and compression force derived from bending at the end of rotation. Surprisingly, immunoblot analysis of PATL3, TREPH2, and ATEH2 phosphosites revealed that a single 24-rpm inversion sufficed to alter phosphorylation within 22 ± 1 s. Repetitive inversions (9 rotations) did not further enhance phosphorylation (Supplemental Fig. S9), indicating the enhanced centrifugal force loading does not modulate phospho-levels. Inversion-triggered phosphorylation persisted from initial gravitropic responses (20 s) to later stages (7200 s) (Figs. 5 and 6). Sixteen phosphosites detected across both timeframes and treatments (Table 1) suggest a single inversion induces slow phosphorylation peaking at 50 s, coinciding with calcium code 2. Notably, phosphoproteomes associated with calcium codes 1 and 2 may not be exclusively force type-dependent; both likely cooperate to activate vesicle trafficking for PIN protein relocation (Table 1).

Mechanical signals (touch, wind, gravity) profoundly impact plant morphology (6, 52, 73). Inversion combines both centrifugal and gravitational forces in general, while touch/wind may alter tissue orientation relative to gravity vector. Our phosphoproteomic studies support that combinatorial forces trigger phosphorylation on overlapping protein sets (52, 54, 59). For example, CPK1, mitogen-activated kinase kinase 1/2, kinesin-like protein for actin-based chloroplast movement 1 (KIN14A), TREPH1 (WPRa4), TREPH2, calmodulin-binding transcription activator (SR1), and transducin/WD40 repeat-like superfamily protein 1 were upregulated in both touch and inversion treatments, though phosphosites sometimes differed (Table 1), implying shared transduction pathways. It is also possible that the bending or flexing of tissues during touching and inversion processes triggers the phosphorylation of these proteins. PATL3, previously identified as gravity-responsive, appeared in inversion and gravistimulation experiments (Fig. 3; Table 1). Immunoblots of PATL3 and TREPH2 revealed distinct regulation: PATL3 responded strongly to gravity (one 6-rpm inversion), while TREPH2 responded to both gravity and touch (Fig. 5). ATEH2 phosphorylation, specific to 20 s multi-inversion (24 rpm), was enhanced by touch/wind, suggesting that both centrifugal force and other types of forces drives its response. These signals may converge via common pathways from different sensors or be sensed by shared mechanoreceptors.

Phosphoproteomics identified early acting kinases (0–20 s) and vesicle-trafficking regulators (e.g., PI4P5 kinases, ARF-GAP proteins; 20–50 s). Bioinformatic analysis of 30 s upside-down treatment-associated phosphoproteins highlighted enrichment in ion/calmodulin-binding (Fig. 4). Mildew resistance locus O-like protein 2 and ABCG36 interact with calmodulin (74, 75), while ATEH2 harbors a calcium-binding EF-hand motif (76). ZAC promotes gravitropic bending by antagonizing Ca^2+^-activated EHB1 (77), with their asymmetric ratio directing auxin flux (71). We observed upregulated pS155-ZAC phosphorylation after 30 s upside-down hold (Table 1), warranting investigation into whether calcium code 2 activates ZAC and if Ca^2+^ mediates its phosphorylation.

Gravitropism involves complex signaling, with the LAZY protein family playing a central role (78). MAPK-mediated LAZY phosphorylation enhances its association with amyloplasts and TOC complexes, and MAPKK inhibition disrupts root gravitropism (79). PIN proteins, acting downstream of LAZY, regulate auxin flux *via* phosphorylation—for example, PIN2 phosphomimetics rescue mutants (50). PIN1 conformational changes, catalyzed by Pin1At, depend on phospho-Ser/Thr-Pro motifs (80), while PID/WAG-mediated PIN3 phosphorylation enables polar rearrangement (81). Our data align with this: MAPK substrates (MASS1/2) increased 16.5-fold after 20 s inversion, and mitogen-activated kinase kinase 1/2 appeared in inversion/gravistimulation assays (Table 1). Novel kinases (CPK1, RAF15, MRK1, PBL8, K9L2.5) emerged within 20 s of reorientation, suggesting their roles in PIN relocation.

**Fig. 7:**
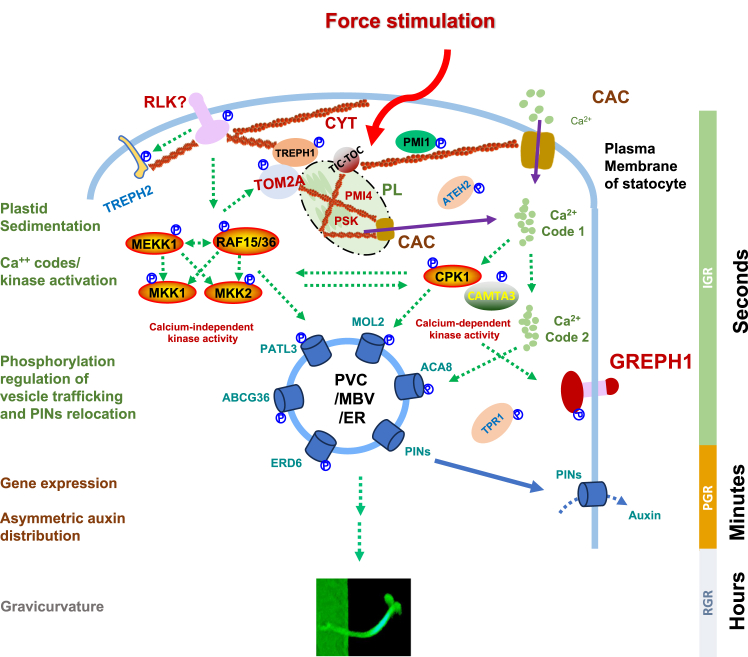
**A model of gravity-sensing mediated by phosphoprotein network.** CAC Ca2+ Ca2+ Code 1 Ca2+ Code 2 PINs plasma membrane of statocyte GREPH1 auxin IGR P PGR RGR seconds minutes hours the plant shoot gravitropism consists of five steps: (1) gravity forces-sensing initiated by plastid (PL, or statolith) sedimentation through endomembrane system, (2) activation of biphasic calcium spikes and receptor-like kinases by plastid movement, (3) phosphorylation regulation of vesicle trafficking that contains PIN proteins, (4) asymmetric distribution of auxin hormone, and (5) finally gravicurvature response of shoot. The conversion of physical force signal to biochemical events (step 1, 2, and 3) is defined as instant gravitropic response (IGR), the gene expression and asymmetric distribution of auxin as the prompt gravitropic response (PGR), and the gravicurvature as the rapid gravitropic response (RGR). Both plasma membrane–bound and plastid membrane–bound calcium channels (CAC) and unknown receptor-like kinase (RLK) might act as mechanosensors. The plastoskeletal protein PMI4 (FtsZ1) forms Z-rings and interacts with the cytosolic and cytoskeletal system containing TREPH1 (WPR4a) to establish a cytoskeleton (CYT)-plastoskeleton (PSK)-continuum (CPC). This continuum triggers both plastidic and plasma membrane–bound Ca²⁺ sensor activation and phosphorylation kinase activation upon reorientation of plant organs. The gravistimulation-responsive rapid increase in cytosolic Ca²⁺ concentration leads to the further activation of calcium-dependent protein kinases and calmodulin. Plastid sedimentation signal is mediated by both calcium-dependent protein kinases, along with the MEKK, RAF15, WIRK1 (RAF36) kinases-mediated phosphorylation events. These calcium-dependent and calcium-independent phospho-relay events regulate downstream transcription factors and vesicle trafficking that relocates PIN proteins to regulate auxin asymmetric redistribution, leading to shoot curvature. Consequently, phytohormones such as jasmonic acid (JA), auxin, and ethylene (ET) participate in the gravitropic response. *Red arrows* mark the organ reorientation stimulation. *Dashed green arrows* stand for putative signaling pathways. *Blue and purple arrows* mark the PIN protein relocation and calcium flux, respectively. TOM2A may function as a plastid movement receptor. PVC, prevacuolar compartment; MBV, multivesicular body; ER, endoplasmic reticulum; TREPH, touch-regulated phosphoprotein; ATEH2, *Arabidopsis thaliana* EF-hand protein 2.

Integrating our 4C phosphoproteomics with published data, we propose a gravity response model (Fig. 7). Upon reorientation beyond the gravitropic setpoint angle (8), plastid sedimentation/movement exerts tension on the cytoskeletal-plastoskeletal continuum (*e.g.*, PMI4, PMI1, TREPH1 protein network). Ion channels Mid1-complementing activity proteins, MscS-like proteins, piezo 1/2 and receptor-like kinases tethered to cytoskeletal-plastoskeletal continuum activate, initiating Ca^2+^-dependent and Ca^2+^-independent phospho-relays that drive PIN relocation and asymmetric auxin distribution, culminating in gravicurvature. Alternatively, plastids may directly engage endomembrane or plasma membrane-localized receptors (e.g., TOM2A, TREPH2, GREPH1) to convert the physical signal of plastid movement to biochemical signals. Genetic screening identified GREPH1—an integrin-like protein—as a novel gravitropism regulator (Figs. 6 and 7), potentially acting as a plastid-movement receptor that activates kinases. This is supported by enrichment of defense-response terms in gravistimulation phosphoproteins (Figs. 3 and 4), possibly reflecting ancient symbiosis-derived mechanisms where plastid–membrane interactions trigger signaling, explaining TOM2A and GREPH1 phosphorylation.

## Data Availability

All MS data have been deposited at the ProteomeXchange Consortium via PRIDE with the table identifier (Username: reviewer_pxd058105@ebi.ac.uk; Password: r7ifsu1isgYb).

## Supplemental Data

This article contains supplemental data.

## Conflict of Interest

The authors declare no competing interests.
