# Supplementary material for: Deciphering the Protein Phosphorylation Dynamics Triggered by Seconds of Force Stimulation
Source: Mol Cell Proteomics. 2026 Feb 19;25(4):101532. doi: 10.1016/j.mcpro.2026.101532 (PMC13054138; doi:10.1016/j.mcpro.2026.101532)
Supplement: Supplemental Figures [file mmc8.pdf]

Supplemental FIG. 1

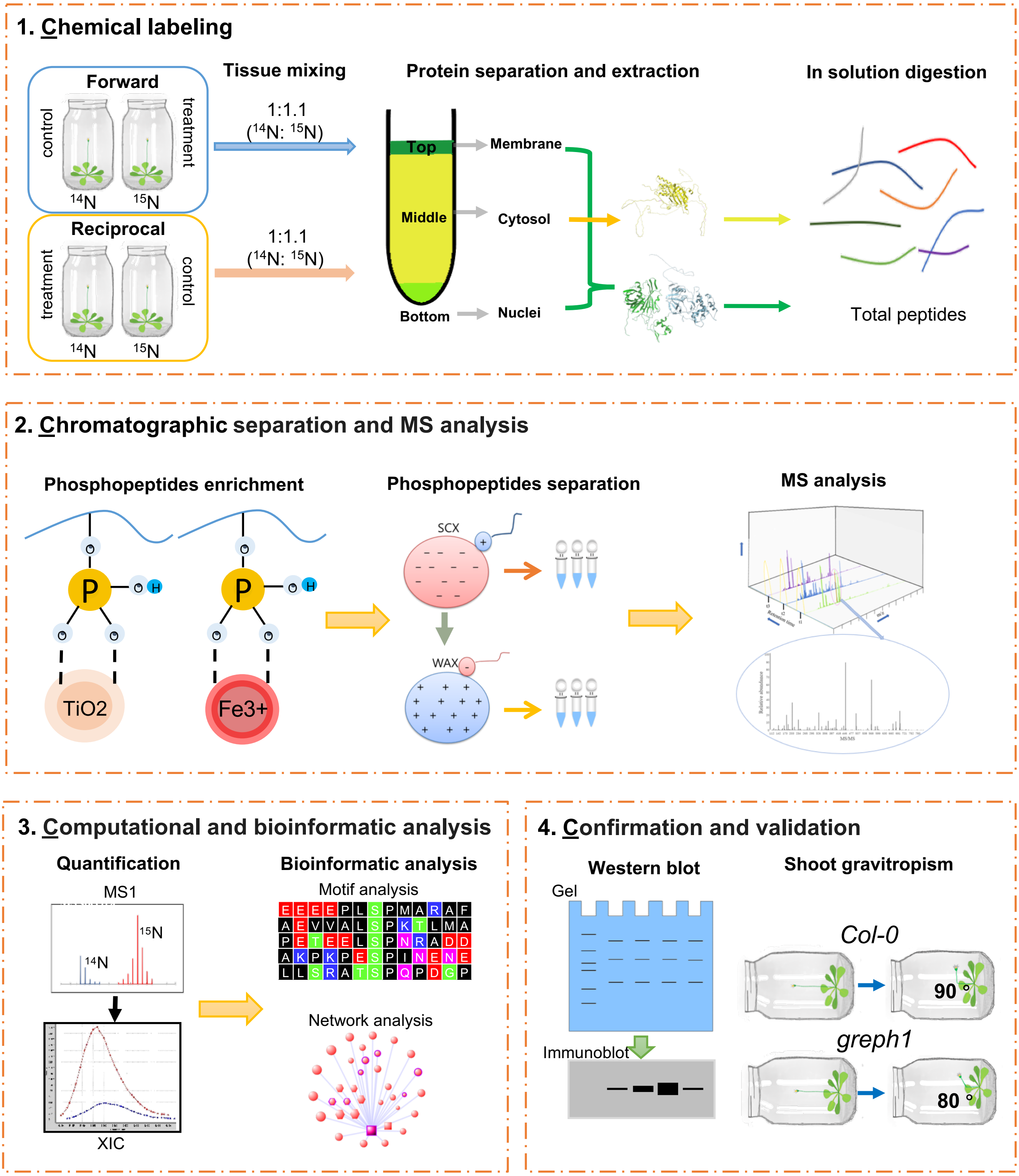

Supplemental FIG. S1. The workflow of SILIA-based and 4 C quantitative phosphoproteomics. The 1st C stands for *in vivo* stable isotope (heavy and light nitrogen) labeling in *Arabidopsis* in this study. After treatment and mixing as described in Materials and Methods, the extracted proteins were separated into membrane and cytoplasmic fractions and then subjected to peptide digestion separately. The 2nd C represents chromatographic separation and MS analysis of the phosphopeptides, which were enriched from total peptides through three rounds of TiO_2 enrichment and one round of Fe^{3+}-IMAC enrichment. The 3rd C means the computational and bioinformatic analysis of the phosphopeptides. The 4th C indicates confirmation and validation of proteomic results using molecular biology methods.

Supplemental FIG. S2

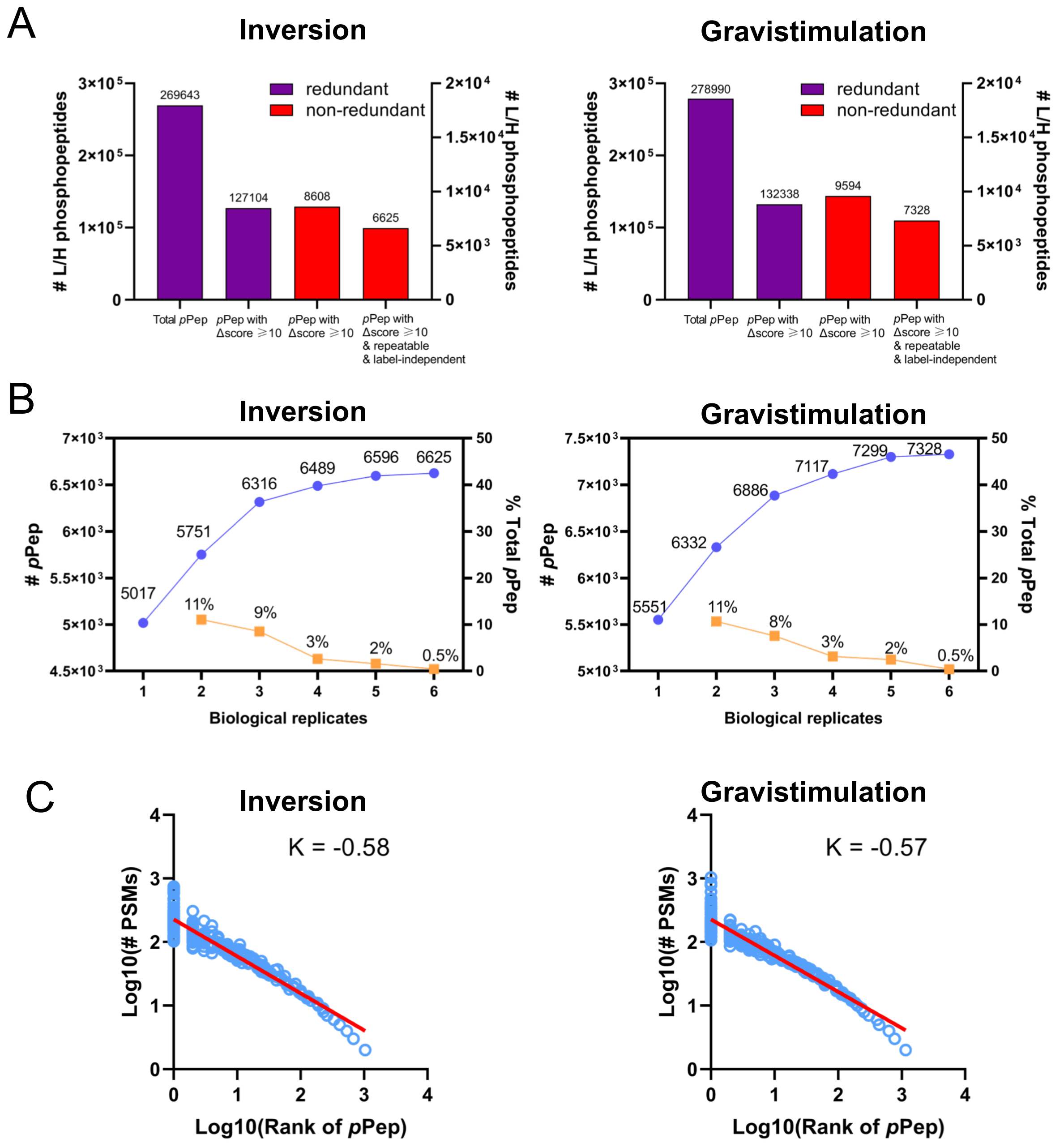

Supplemental FIG. S2. Proteomic analysis of total identified phosphopeptides in this study. *A,* the bar represents the numbers of ambiguous, redundant, non-repeatable, and label-dependent phosphopeptides of an FDR <0.01, the numbers of unambiguous, redundant, non-repeatable, and label-dependent phosphopeptides of both FDR <0.01 and delta score ≥10, the numbers of unambiguous, non-redundant, non-repeatable and label-independent phosphopeptides of both FDR <0.01 and delta score ≥10 and the numbers of unambiguous, non-redundant, repeatable and label-independent phosphopeptides of both FDR < 0.01 and delta score ≥ 10 for both inversion (left) and gravistimulation experiments (right), respectively (Supplemental Table S2, S3). *B*, the blue curves represent the accumulation of phosphopeptides while the yellow curves represent the cumulating contribution over 6 biological replicates for two sets of experiments. The left plot shows the results of the inversion experiment and the right plot presents the results of gravistimulation experiment. *C*, distribution of the PSM numbers of each phosphopeptide over the rank (or index) of the entire population of phosphopeptides for the inversion experiment (left) and gravistimulation experiment (right). The k represents the slope of the fitting curve of the rank-frequency distribution by Zipf's law.

Supplemental FIG. S3

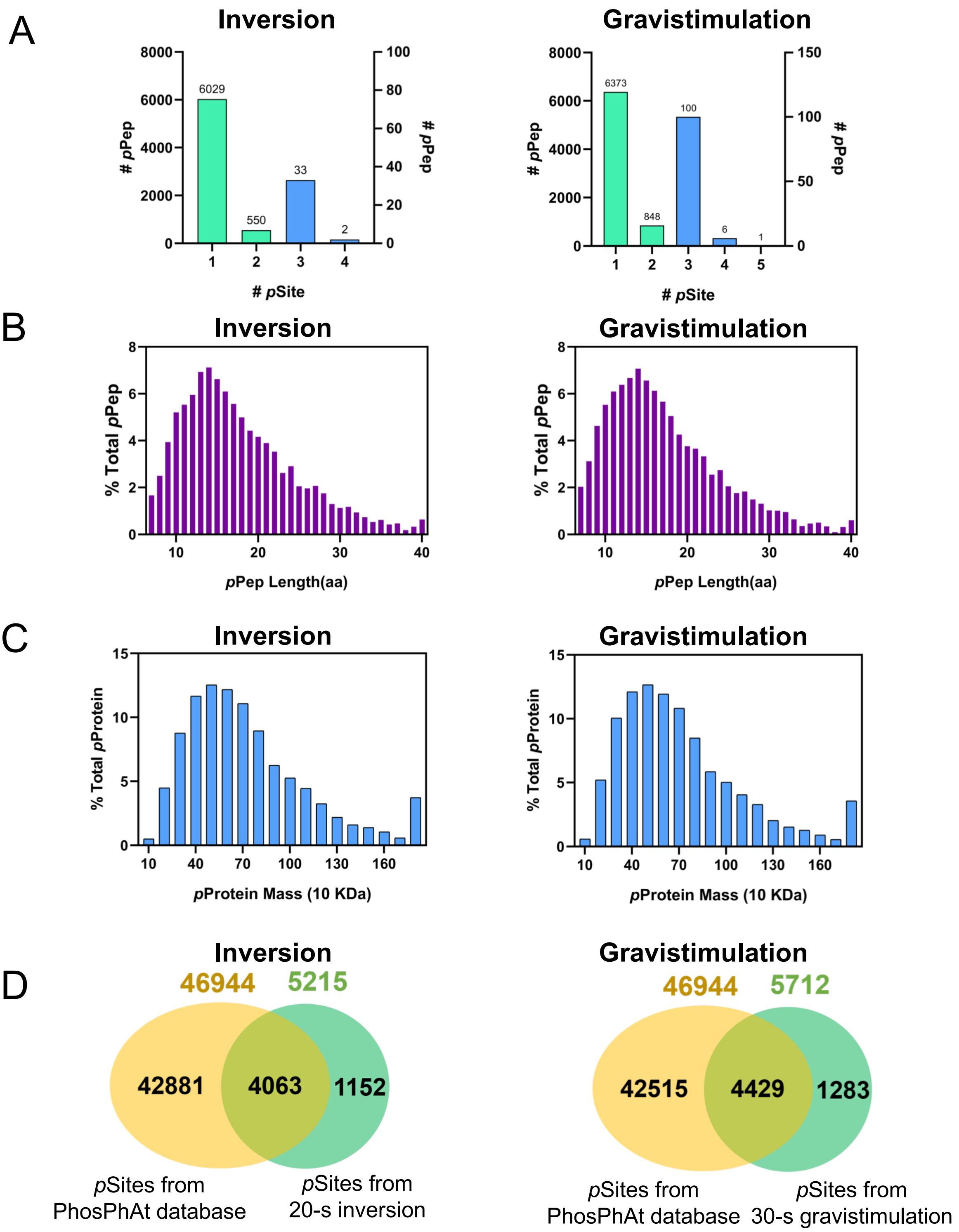

Supplemental FIG. S3. Bioinformatic analysis of identified phosphopeptides and phosphoproteins in this study. *A*, distribution of phosphopeptide subpopulations with specific numbers of phosphosite on phosphopeptides for two sets of experiments. The pSite stands for phosphosite. *B*, the distribution of the identified phosphopeptides over their primary lengths for two sets of experiments. C, the distribution of the molecular weight of identified leading phosphoproteins for two sets of experiments. The *p*Protein stands for phosphoprotein. *D,* venn diagrams show the overall phosphosites deposited in the repository database of PhosPhAt and identified from the Inversion group and Gravistimulation group, respectively. In this figure, the results for the Inversion group are on the left, and the analysis for the gravistimulation group is on the right. (Supplemental Table S2, S3).

Supplemental FIG. S4

A

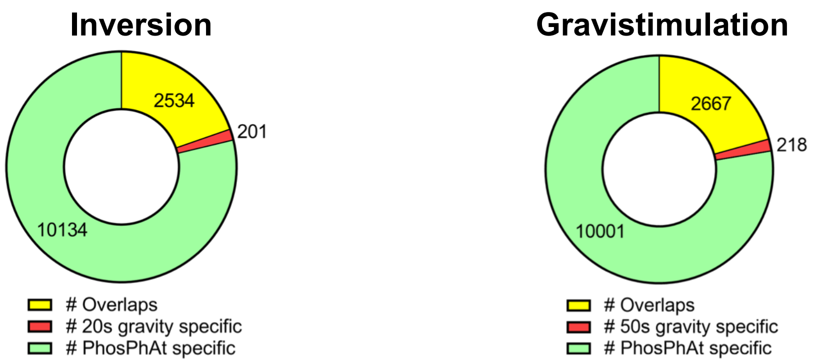

Supplemental FIG. S4. Bioinformatic analysis of identified phosphoproteins in this study. *A*, the fan plots show the number of phosphoproteins found in the PhosPhAt database and Inversion experiment (left) or Gravistimulation experiment (right). (Supplemental Table S2, S3).

Supplemental FIG. S5

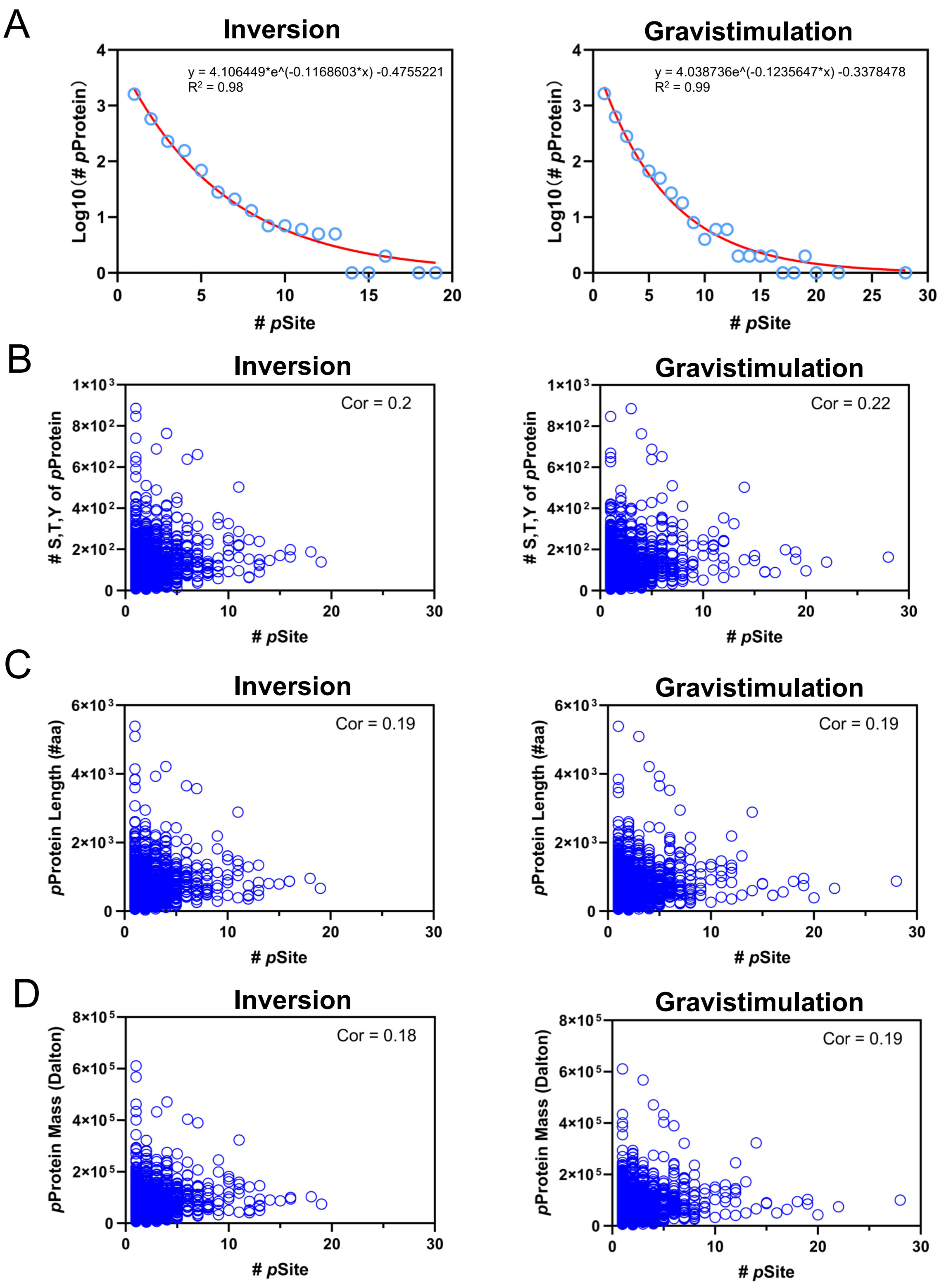

Supplemental FIG. S5. Bioinformatic analysis of identified phosphoproteins from the Inversion group and the Gravistimulation group. *A*, the power law equations of the fitting curve describe the correlation in between the total number of phosphoproteins and the specific number of phosphorylation site for the two sets of experiments. *R*2 represents the coefficient of the curve determination. *B,* the correlation in between the number of amino acid (S, serine; T, threonine; Y, tyrosine) and a specific number of phosphosites of phosphoproteins for two sets of experiments. The Cor represents the Pearson correlation number. *C*, the correlation in between the primary amino acid (aa) sequence length and the specific number of phosphosite of a phosphoprotein for two sets of experiments. The Cor represents the Pearson correlation number. *D,* the correlation in between the molecular weight (monoisotopic mass) and the specific number of phosphosite of a phosphoprotein for two sets of experiments. The Cor represents the Pearson correlation number. (Supplemental Table S2, S3).

Supplemental FIG. S6

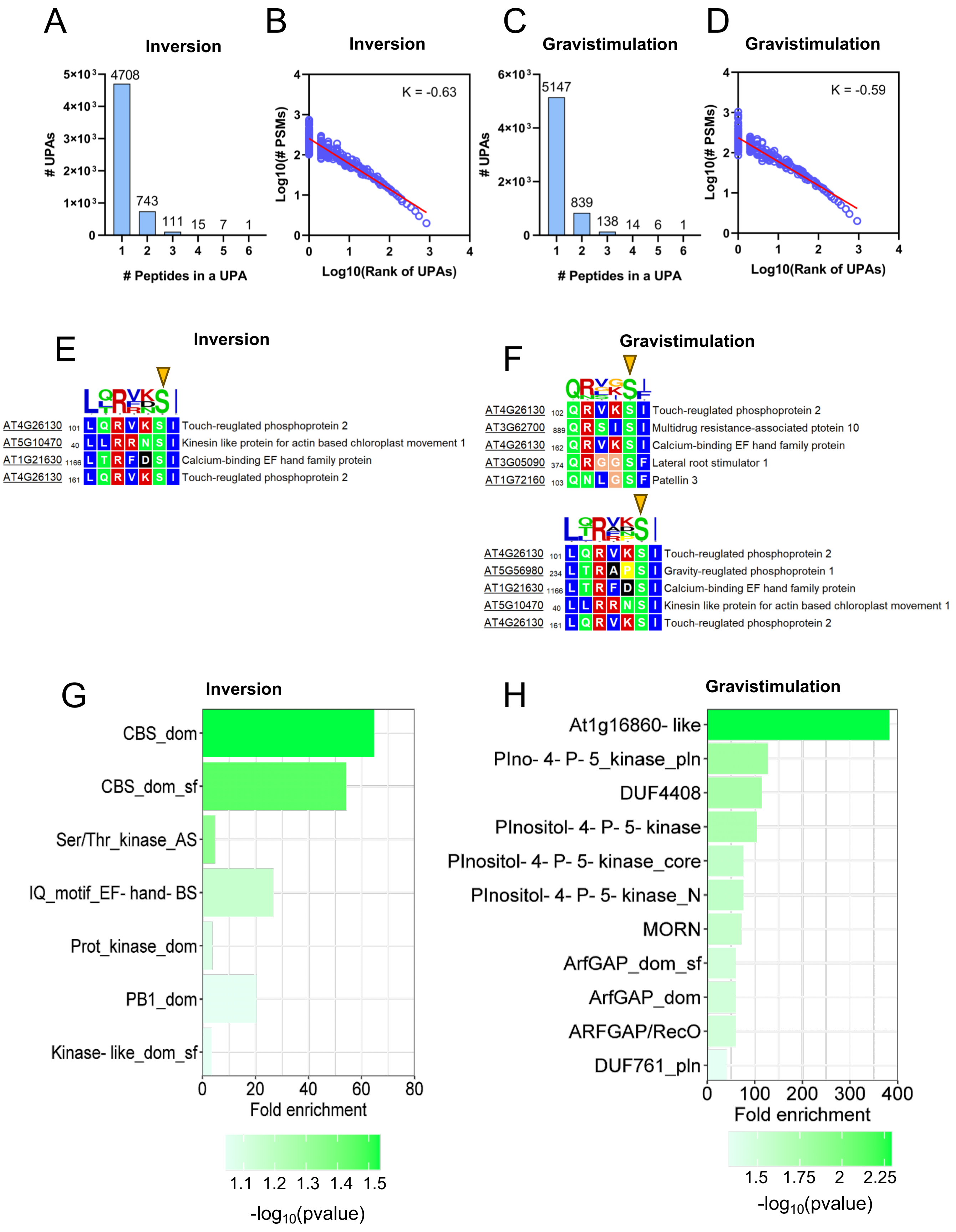

Supplemental FIG. S6. Bioinformatic analysis of significantly regulated phosphoproteins by both inversion and gravistimulation treatments. *A*, the phosphopeptide groups under inversion stimulation were distributed and classified according to the number of phosphopeptides present in a UPA. *B*, the rank frequency distribution of PSM numbers on the UPA index according to Zipf's law for the inversion stimulation experiment. *C*, the phosphopeptide groups under gravistimulation were distributed and classified according to the number of phosphopeptides present in a UPA. *D*, rank frequency distribution of PSM numbers on the UPA index according to Zipf's law for gravity stimulation experiment. *D*, phosphorylation site motifs constructed using Motif-X were derived from a set of significantly inversion upregulated phosphopeptides. Yellow triangles indicate phosphorylation sites. *E*, phosphorylation site motifs constructed using Motif-X were derived from a set of significantly gravity stimulation upregulated phosphopeptides. Yellow triangles indicate phosphorylation sites. *G* - *H*, interPro domains are enriched in phosphorylated proteins upregulated by inversion stimulation (*G*) and 30-s gravistimulation (*H*), respectively. (Supplemental Table S4 and S5).

Supplemental FIG. S7

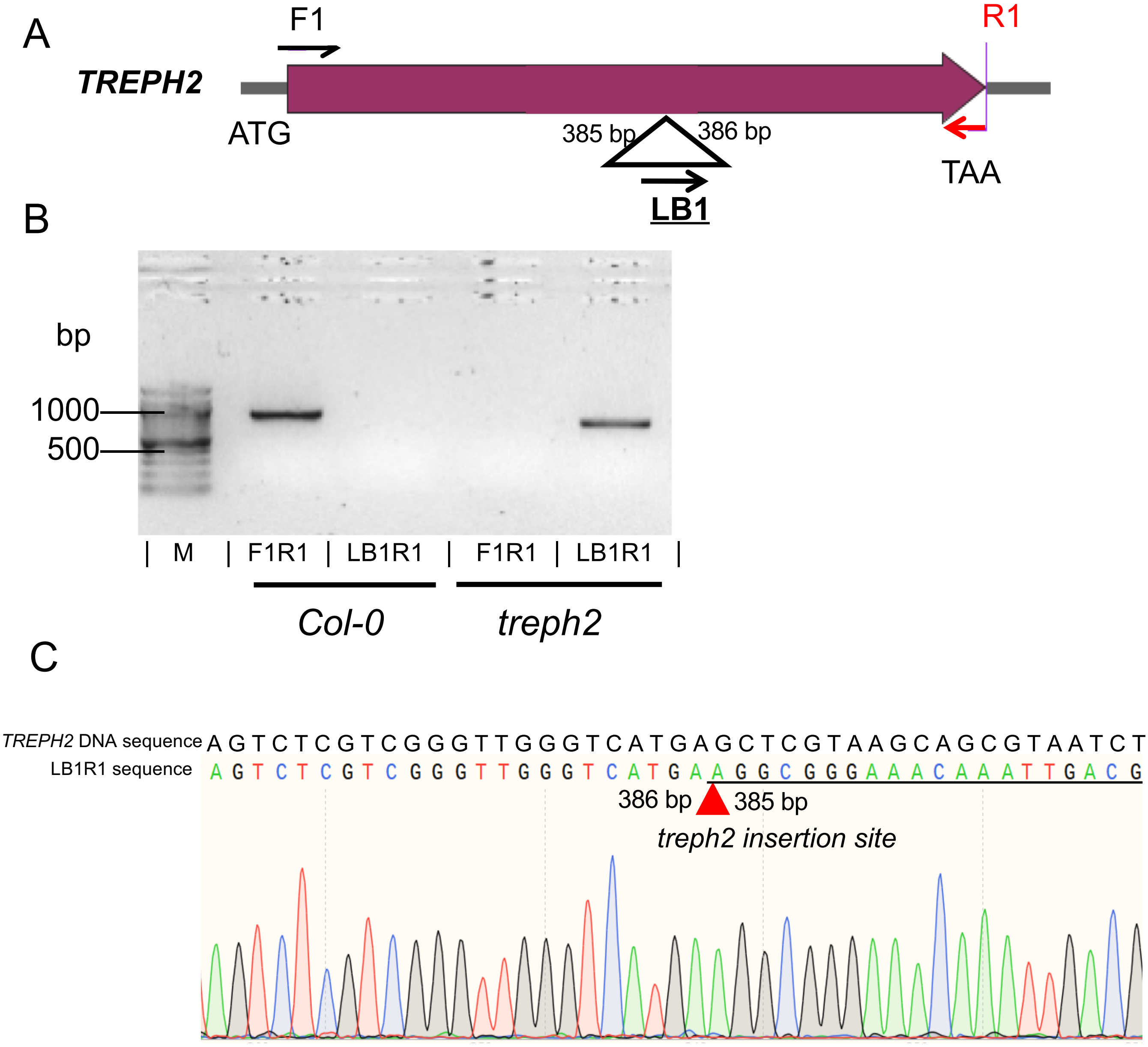

Supplemental FIG. S7. Genotyping of T-DNA insertional mutant. *A*, DNA schematic representation of TREPH2. Triangles represent T-DNA insertion sites in T-DNA insertional mutants. Primers used for genotyping are presented with arrows. Black and red arrows stand for forward and reverse primers, respectively. Left T-DNA border primers were highlighted with bold and underlined. Solid line, 5`- and 3`-UTR. Purple box, exon region. ATG and TAA indicate the start and stop codon, respectively. *B*, Genotyping of T-DNA insertions by PCR. Primer pairs used to amplify each fragment were LB1 + R1 (LR1 fragment) and F1 + R1 (F1R1 fragment), respectively. The size of the FR fragment is 859 bp. *C*, T-DNA insertion site verification of *treph2* by DNA sequencing. The insertion site at 385 bp of TREPH2 gene.

Supplemental FIG. S8

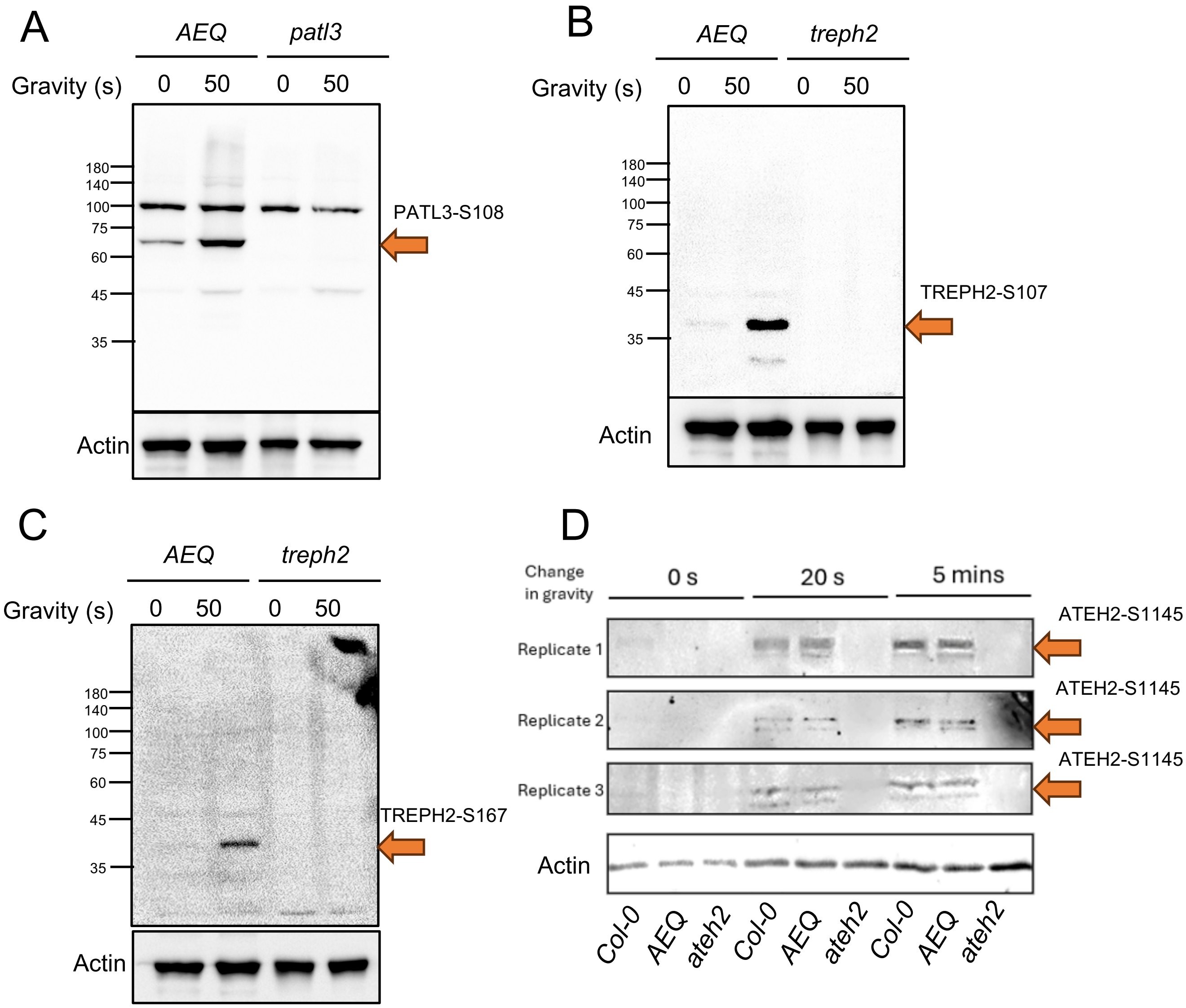

Supplemental FIG. S8. Antibody specificity validation. *A*, negative control for *p*PATL3 phosphorylation antibody in the gravity-stimulated mutant *patl3* compared to *AEQ*. *B, C*, negative control for *p*TREPH2-S107 (*B*) and *p*TREPH2-S167 (*C*) phosphorylation antibodies in the gravity-stimulated mutant *treph2* compared to *AEQ*, respectively. *D*, negative control for *p*ATEH2 phosphorylation antibody in the gravity-stimulated mutant *ateh2* compared to *Col-0 and AEQ*.

Supplemental FIG. S9

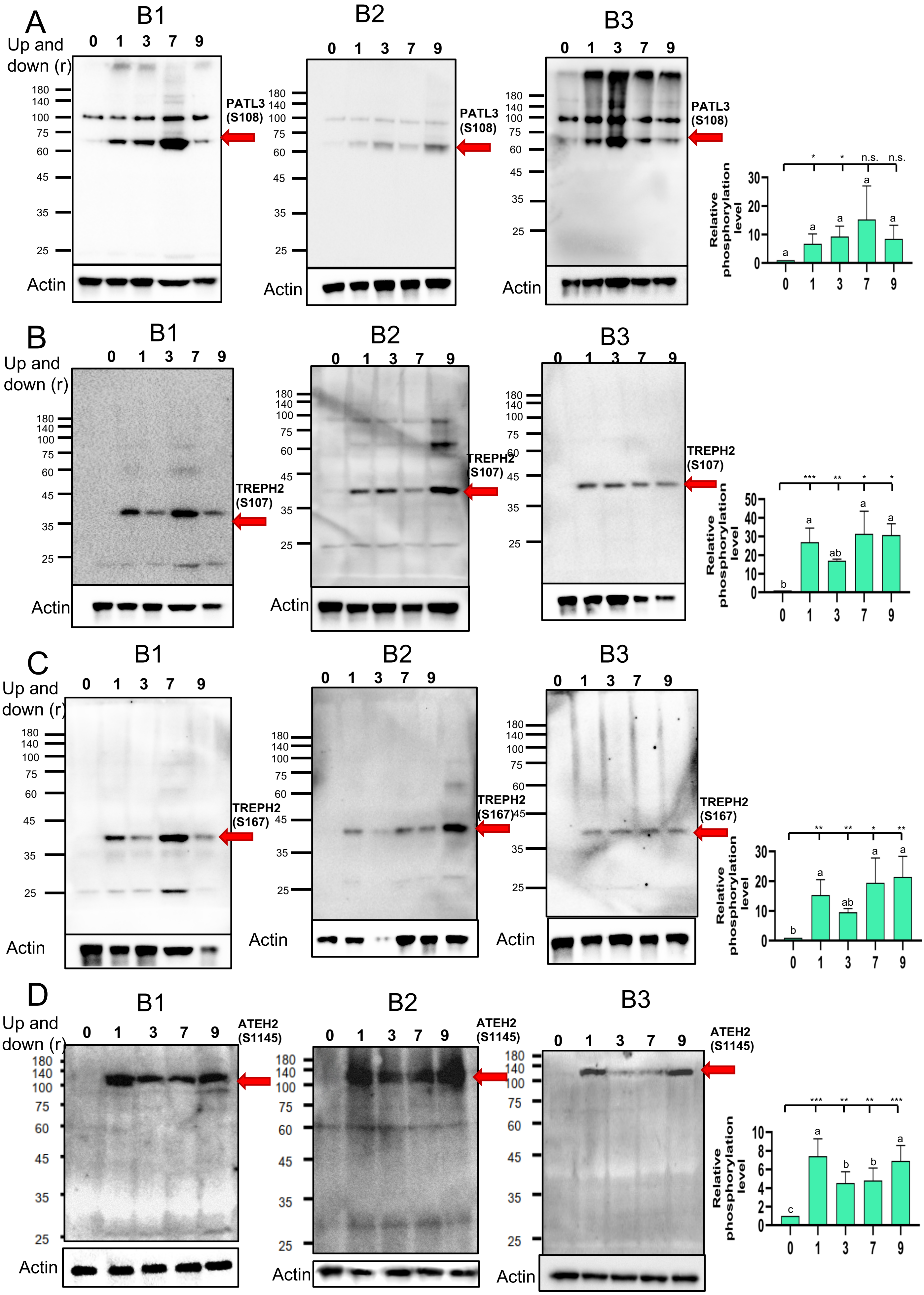

Supplemental FIG. S9. Western blot analysis of phosphorylated proteins after different rounds of gravity stimulation. *A*, the phosphorylation level of PATL3 was enhanced after different rounds of inverted gravity stimulation in three biological replicates. *B*, the phosphorylation level of TREPH2-S107 was enhanced after different rounds of inverted gravity stimulation in three biological replicates. *C*, the phosphorylation level of TREPH2-S167 was enhanced after different rounds of inverted gravity stimulation in three biological replicates. *D*, the phosphorylation level of ATEH2-S1145 was enhanced after different rounds of inverted gravity stimulation in three biological replicates. For all graphs in this figure, the relative levels of enhancement were measured by Image J and presented as bar graphs of three biological replicates on the right side of each figure. The data were analyzed by Student's t test; n.s. represents $P > 0.05$; *, **, *** represent $0.01 < P < 0.05$, $0.01 < P < 0.001$, and $P < 0.001$, respectively. Different letters indicate significant differences at the 5% level shown above each bar, based on Tukey's range test.

Supplemental FIG. S10

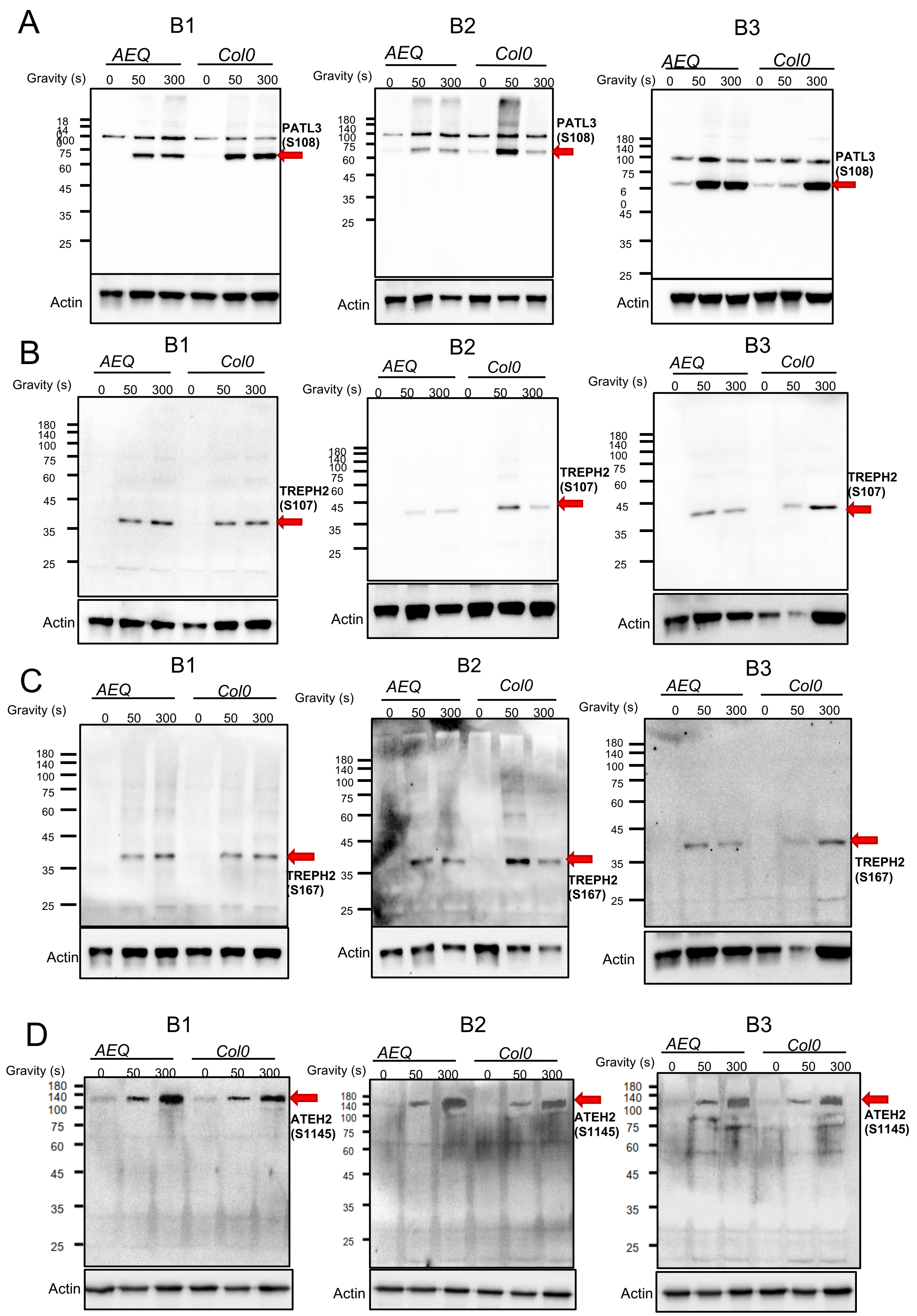

Supplemental FIG. S10. Immunoblotting analysis of phosphorylation of gravity-upregulated phosphoproteins. *A*, immunoblotting of whole protein gels demonstrated gravity-enhanced phosphorylation of S108 of PATL3 in *Col-0* and *AEQ* transgenic plants in three biological replicates. *B*, immunoblotting of whole protein gels demonstrated gravity-enhanced phosphorylation of S107 of TREPH2 in *Col-0* and *AEQ* transgenic plants in three biological replicates. *C*, immunoblotting of whole protein gels demonstrated gravity-enhanced phosphorylation of S107 of TREPH2 in *Col-0* and *AEQ* transgenic plants in three biological replicates. *D*, immunoblotting of whole protein gels demonstrated gravity-enhanced phosphorylation of S1145 of ATEH2 in *Col-0* and *AEQ* transgenic plants in three biological replicates. Plants were inverted 180-degree for one time and hold down for 50s or 300s.

Supplemental FIG. S11

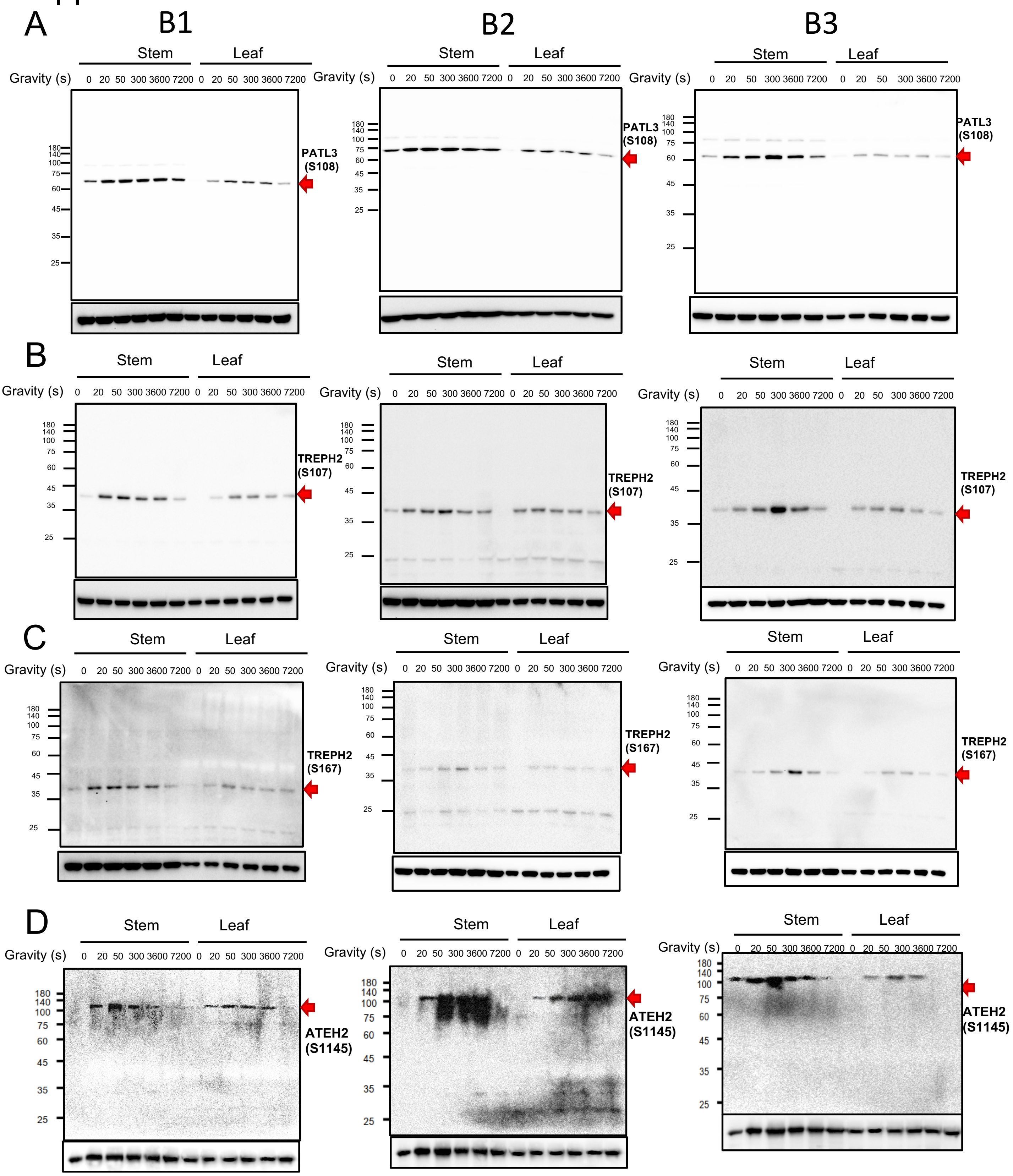

Supplemental FIG. S11. Immunoblotting results of temporal changes in phosphorylation levels after gravity vector changes in different parts of adult plants. *A* - *D*, immunoblotting results showed that in three biological replicates, the phosphorylation levels of PATL3-S108 (*A*), TREPH2-S107 (*B*), TREPH2-S167 (*C*), and ATEH2-S1145 (*D*) in stem and leaf parts changed over time under gravity stimulation. Total proteins were extracted from stem and leaf parts of 25-day-old adult *Arabidopsis* plants.

Supplemental FIG. S12

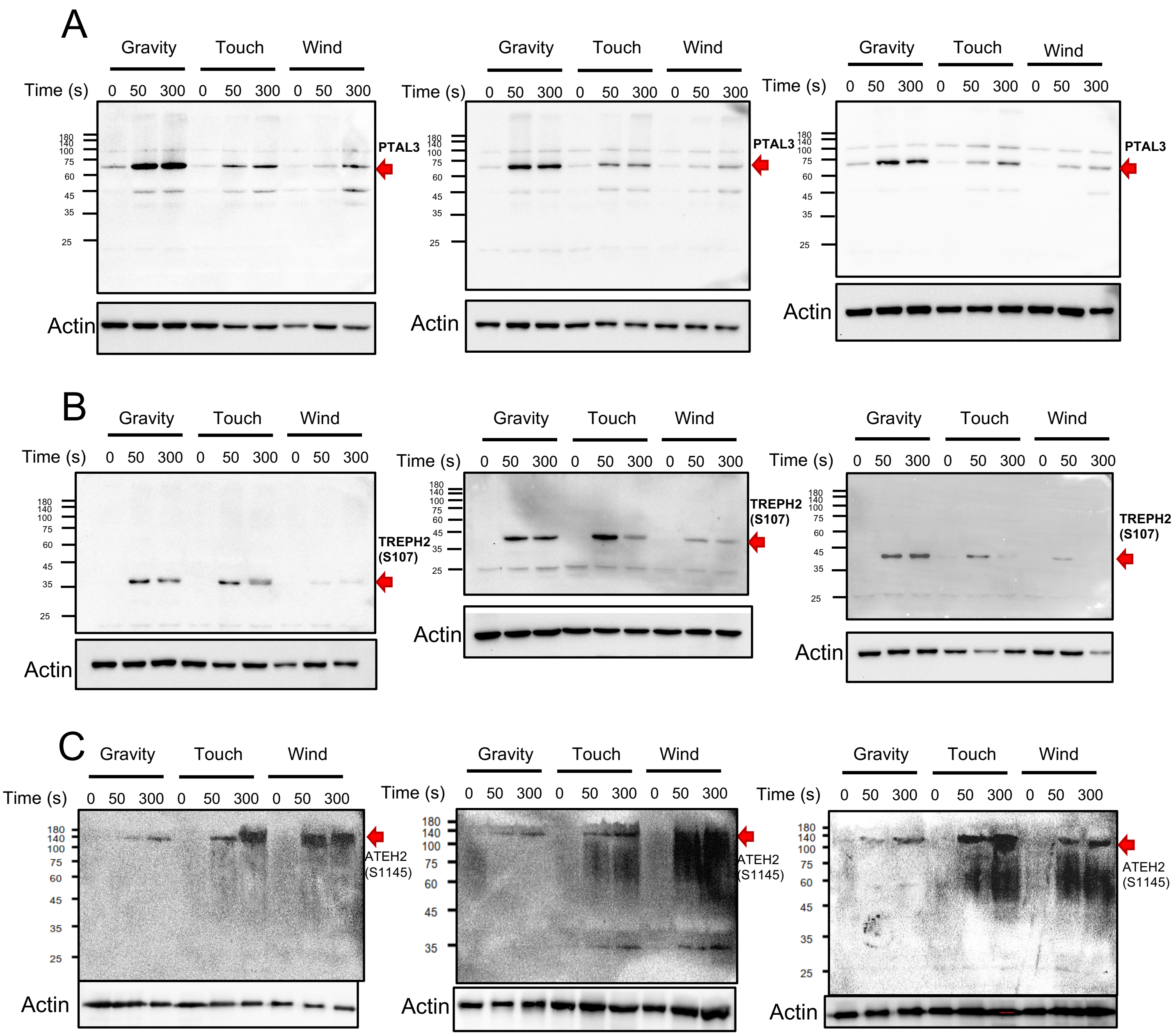

Supplemental FIG. S12. Changes in the phosphorylation level of gravity-stimulated phosphoproteins under different types of stimulation. *A*, changes in the phosphorylation level of PATL3-S108 under different types of stimulation. *B*, changes in the phosphorylation level of TREPH2-S107 under different types of stimulation. *C*, changes in the phosphorylation level of ATEH2-S1145 under different types of stimulation. Three biological replicates were conducted for each antibody. Total protein was extracted from the aerial parts of 25-day-old adult *Arabidopsis* plants.

Supplemental FIG. S13

Gravicurvature assay on the light-grown Arabidopsis seedling stem (The stem gravicuvature of selected 17 mutants was recorded 24h after a 90-degree rotation)							
		**Replicate 1**			**Replicate 2**		
**Mutant/ genotype**	**Accession number**	**Average bending angles**	**T. Test vs. *AEQ***	**T.Test vs. *Col-0***	**Average bending angles**	**T.Test vs. *AEQ***	**T.Test vs. *Col-0***
Col-0		47.36	4.514E-01	1.000E+00	45.02	1.992E-01	1.000E+00
AEQ		46.19	1.000E+00	4.514E-01	42.93	1.000E+00	1.991E-01
greph1 /AEQ	**AT5G56980**	**9.34**	**5.357E-28**	**4.333E-37**	**15.90**	**2.126E-14**	**1.541E-24**
erd6 mlo2 /AEQ	AT1G08930; AT1G11310	21.88	2.298E-20	3.433E-23	22.24	1.071E-11	2.528E-16
erd6 aca8 /AEQ	AT1G08930; AT5G57110	24.27	5.025E-24	7.028E-27	26.87	1.467E-13	4.466E-21
mkk1 /AEQ	AT4G26070	24.05	5.875E-19	3.135E-24	27.83	1.945E-09	1.177E-16
treph1 /AEQ	AT5G55860	31.61	2.233E-06	3.158E-10	29.42	3.112E-05	2.405E-10
mlo2 mlo6 /AEQ	AT1G11310; AT1G61560	29.25	1.084E-06	1.793E-11	31.99	2.657E-03	5.832E-07
aca8 aca10 /AEQ	AT5G57110; AT4G29900	33.60	1.721E-06	2.181E-09	35.05	4.114E-03	5.234E-06
abcg36 aca8 /AEQ	AT1G59870; AT5G57110	39.14	1.397E-04	6.778E-07	41.35	4.086E-01	1.668E-02
aca8 mlo2 /AEQ	AT5G57110; AT1G11310	39.73	6.868E-04	8.101E-06	42.05	6.810E-01	8.865E-02
erd6 abcg36 /AEQ	AT1G08930; AT1G59870	41.82	6.251E-02	3.997E-03	42.58	8.859E-01	1.899E-01
abcg36 abcg29 /AEQ	AT1G59870; AT3G16340	44.14	3.283E-01	7.055E-02	42.96	9.869E-01	2.248E-01
mlo2 ateh2 /AEQ	AT1G11310; AT1G21630	44.63	4.190E-01	1.243E-01	43.81	6.569E-01	4.508E-01
treph2 /AEQ	AT4G26130	46.20	9.961E-01	5.502E-01	44.85	4.515E-01	9.275E-01
erd6 ateh2 /AEQ	AT1G08930; AT1G21630	46.03	9.341E-01	4.273E-01	47.14	4.268E-02	1.739E-01
aca8 ateh2 /AEQ	AT5G57110; AT1G21630	45.22	7.502E-01	3.550E-01	48.50	8.576E-02	1.291E-01
mkk2 /AEQ	AT4G29810	48.52	2.947E-01	5.145E-01	49.05	7.004E-03	1.706E-02
mrk1 mapkkk /AEQ	AT3G63260; AT3G58640	51.69	2.508E-03	7.444E-03	48.02	4.937E-02	1.407E-01

Supplemental FIG. S13. Screening for gravity-responsive mutants under the light. Thise table summarizes the curvature results after changing the gravity vector for different knockout lines. Plants of a total of 20 genotypes were compared for bending stem curvature, with light-grown seedlings placed from upward to 90° for 24 h. The values are the means of one biological replicate consisting of at least 30 plants per genotype. Student's t-test was used in this study

Supplemental FIG. S14

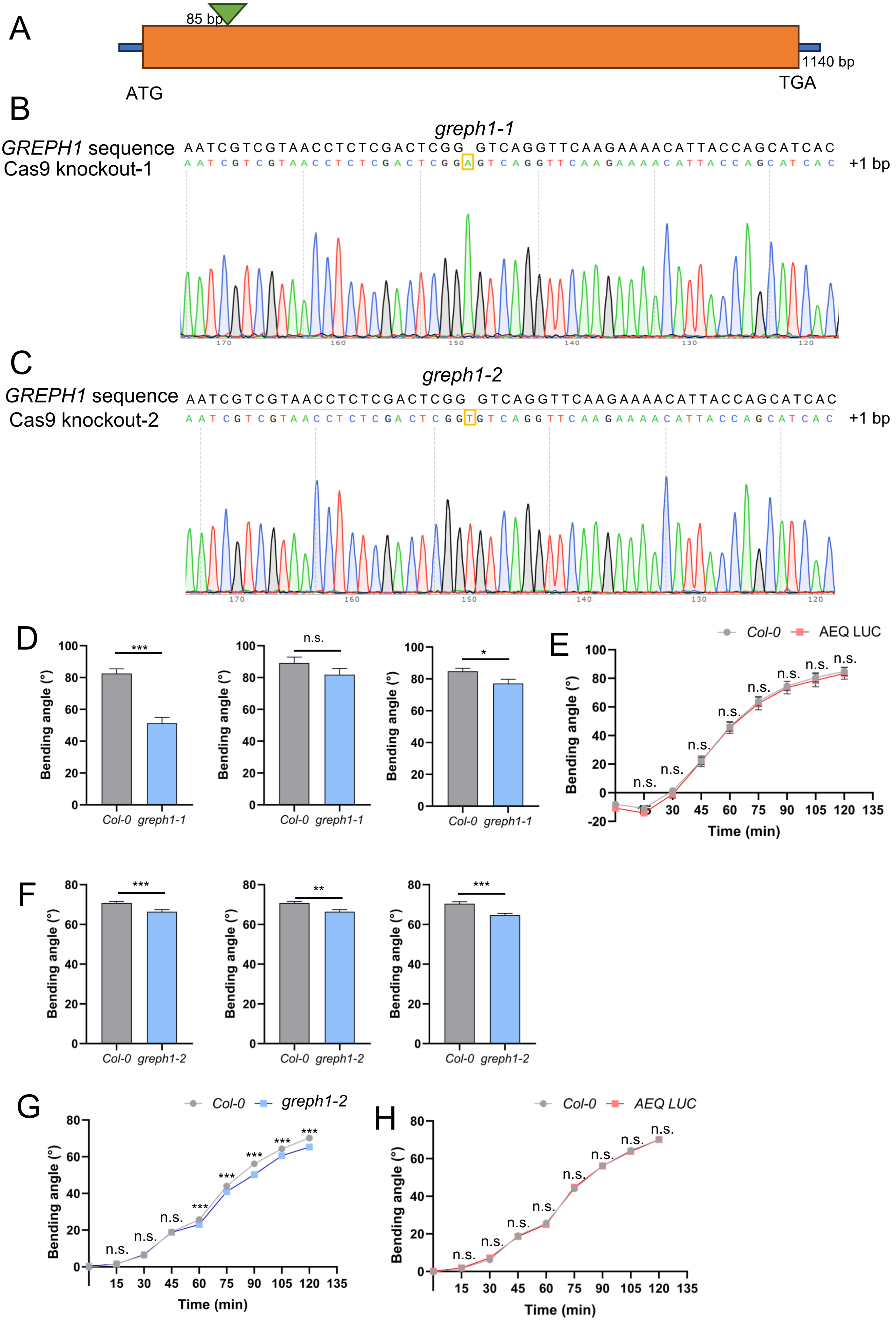

Supplemental FIG. S14. Genotyping and the gravitropic response of *greph1* mutants.

A, Schematic diagram of DNA for GREPH1. The triangle represents the sgRNA target sequence. *B* - *C*, DNA sequencing results for *greph1-1* (*B*) and *greph1-2* (*C*) knock-out mutants. *D*, shoot bending angles after 2 h of gravity vector shift in *greph1-1* and *Col-0*. Three biological replicates were analyzed. *E*, the gravitropic response of *Col-0* and *AEQ*. Values are the average of at least 150 plants per genotype, with angles recorded every 15 min. *F*, shoot bending angles after 2 h of gravity vector shift in *greph1-2* and *Col-0*. Three biological replicates were analyzed. *G*, the gravitropic response of *Col-0* and *greph1-2*. Values are the average of at least 150 plants per genotype, with angles recorded every 15 min. *H*, the gravitropic response of *Col-0* and *AEQ*. Values are the average of at least 150 plants per genotype, with angles recorded every 15 min. The data were analyzed by Student’s t test; n.s. represents $P > 0.05$; *, **, *** represent $0.01 < P < 0.05$, $0.01 < P < 0.001$, and $P < 0.001$, respectively.

Supplemental FIG. S15

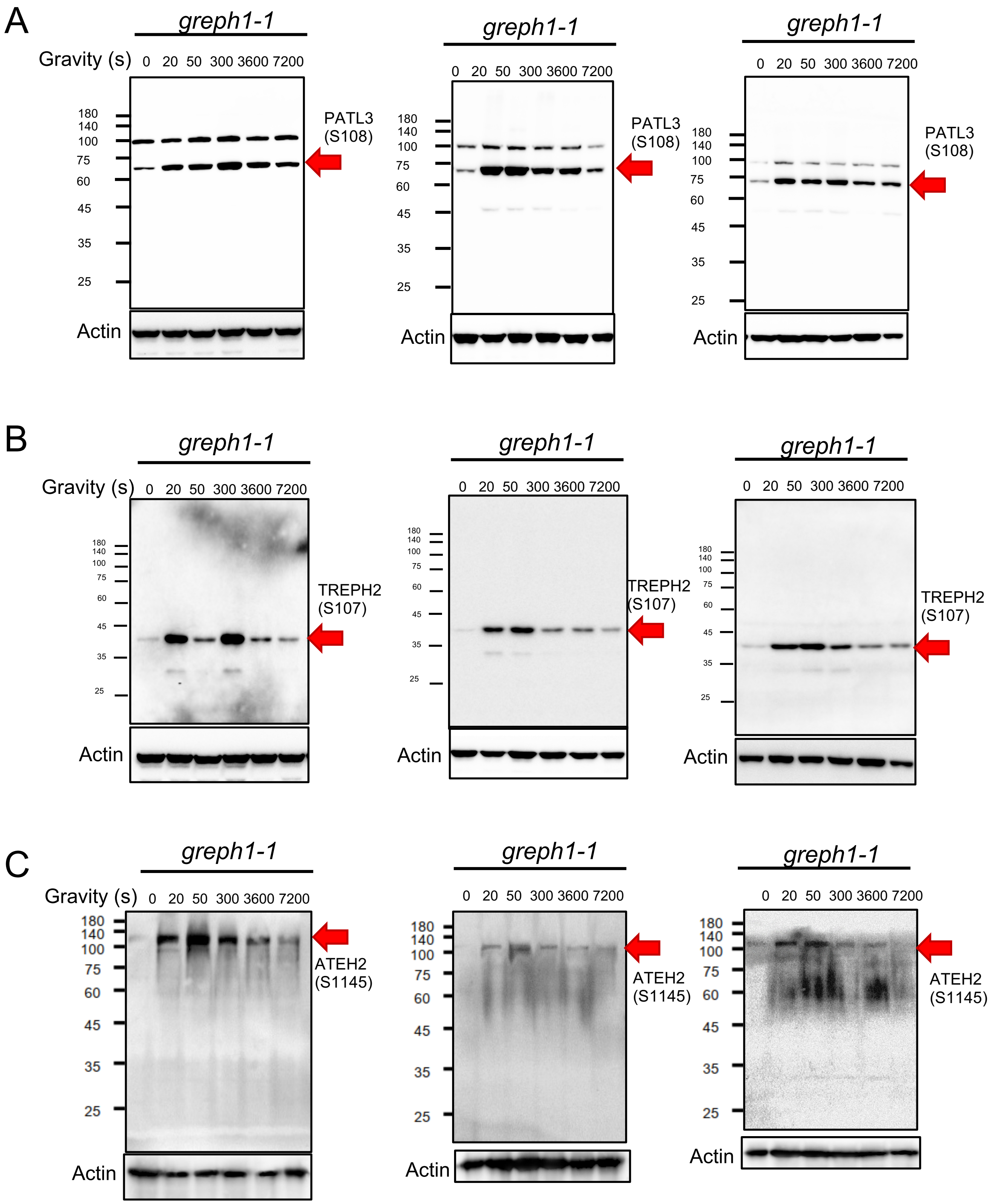

Supplemental FIG. 15. Immunoblotting results showed the temporal changes in phosphorylation levels after gravity vector change in *greph1-1*. *A - C*, immunoblotting results showed that in three biological replicates, the phosphorylation levels of PATL3-S108 (*A*), TREPH2-S107 (*B*), and ATEH2-S1145 (*C*) changed over time under gravity stimulation. Total protein was extracted from the shoot parts of 25-day-old adult *Arabidopsis* plants.
